# Neurofibromin 1 mutations impair the function of human induced pluripotent stem cell-derived microglia

**DOI:** 10.1242/dmm.049861

**Published:** 2023-12-11

**Authors:** Leonard D. Kuhrt, Edyta Motta, Nirmeen Elmadany, Hannah Weidling, Raphaela Fritsche-Guenther, Ibrahim E. Efe, Olivia Cobb, Jit Chatterjee, Lucy G. Boggs, Marina Schnauß, Sebastian Diecke, Marcus Semtner, Corina Anastasaki, David H. Gutmann, Helmut Kettenmann

**Affiliations:** ^1^ Cellular Neurosciences, Max-Delbrück-Center for Molecular Medicine in the Helmholtz Association, 13125 Berlin, Germany; ^2^ Technology Platform Pluripotent Stem Cells, Max-Delbrück-Center for Molecular Medicine in the Helmholtz Association, 13125 Berlin, Germany; ^3^ Charité – Universitätsmedizin Berlin, Berlin, Germany; ^4^ Department of Neurosurgery, University Medical Center Schleswig-Holstein, 24105 Kiel, Germany; ^5^ German Cancer Consortium (DKTK), Clinical Cooperation Unit (CCU), Neuroimmunology and Brain Tumor Immunology, German Cancer Research Center (DKFZ), 69120 Heidelberg, Germany; ^6^ Department of Neurology, Medical Faculty Mannheim (MCTN), University of Heidelberg, 68167 Mannheim, Germany; ^7^ Berlin Institute of Health (BIH) at Charité – Universitätsmedizin Berlin, BIH Metabolomics Platform, 13353 Berlin, Germany; ^8^ Department of Neurology, Washington University School of Medicine, St. Louis, MO 63110, USA; ^9^ Klinik für Augenheilkunde, Charité – Universitätsmedizin Berlin, 13353 Berlin, Germany; ^10^ Shenzhen Institute of Advanced Technology, Chinese Academy of Sciences, Shenzhen, China, 518000

**Keywords:** Microglia, Neurofibromatosis 1, Human induced pluripotent stem cells, Purinergic receptors, Phagocytosis, Motility

## Abstract

Neurofibromatosis type 1 (NF1) is an autosomal dominant condition caused by germline mutations in the neurofibromin 1 (*NF1*) gene. Children with NF1 are prone to the development of multiple nervous system abnormalities, including autism and brain tumors, which could reflect the effect of *NF1* mutation on microglia function. Using heterozygous *Nf1*-mutant mice, we previously demonstrated that impaired purinergic signaling underlies deficits in microglia process extension and phagocytosis *in situ*. To determine whether these abnormalities are also observed in human microglia in the setting of NF1, we leveraged an engineered isogenic series of human induced pluripotent stem cells to generate human microglia-like (hiMGL) cells heterozygous for three different *NF1* gene mutations found in patients with NF1. Whereas all *NF1*-mutant and isogenic control hiMGL cells expressed classical microglia markers and exhibited similar transcriptomes and cytokine/chemokine release profiles, only *NF1*-mutant hiMGL cells had defects in P2X receptor activation, phagocytosis and motility. Taken together, these findings indicate that heterozygous *NF1* mutations impair a subset of the functional properties of human microglia, which could contribute to the neurological abnormalities seen in children with NF1.

## Teaser

**Summary:** Human microglia-like cells heterozygous for three different *NF1* gene mutations found in patients with neurofibromatosis type 1 show defects in P2X receptor activation, phagocytosis and motility.

## INTRODUCTION

Microglia are specialized brain tissue-resident macrophages that derive from the yolk sac and populate the developing central nervous system (CNS) during embryogenesis (Ginhoux et al., 2010; Hoeffel and Ginhoux, 2015). These brain monocytes constantly survey their local environment to ensure homeostasis, as well as change their functional phenotype in the setting of injury and disease. As such, microglia have been implicated as key regulators of neurodevelopmental, neuro-oncological and neurodegenerative disorders, including autism (Derecki et al., 2012; Kana et al., 2019), brain cancer (Hambardzumyan et al., 2016; Pasqualini et al., 2020), and Alzheimer's dementia (Hong et al., 2016; Leyns et al., 2019). For the most part, their critical roles in human CNS disease have largely been explored in murine model systems using pharmacological and genetic approaches (Costa et al., 2021; Hammond et al., 2019; Sierra et al., 2019; Wolf et al., 2017). However, there are notable differences between murine and human microglia (Geirsdottir et al., 2020; Marsh et al., 2022), prompting the development of protocols to generate human microglia (Jairaman et al., 2022; McQuade and Blurton-Jones, 2022; Takata et al., 2017).

With further refinement of the methodologies to produce human induced pluripotent stem cell (hiPSC)-derived microglia-like (hiMGL) cells (Jairaman et al., 2022; McQuade and Blurton-Jones, 2022; Takata et al., 2017), it became possible to define the impact of disease-causing mutations on human microglia function. This is particularly important for neurodevelopmental and cancer predisposition syndromes, such as neurofibromatosis type 1 (NF1), an autosomal dominant condition caused by germline mutations in the human neurofibromin 1 (*NF1*) gene. Previously, we have shown that heterozygous *Nf1*-mutant murine microglia make key contributions to low-grade brain tumor (glioma) pathogenesis (de Andrade Costa et al., 2022; Pan et al., 2018; Toonen et al., 2017; Solga et al., 2015a). To directly explore the impact of *Nf1* mutation on microglia, we previously identified sex-specific defects in microglia from mice heterozygous for a germline inactivating mutation in the murine *Nf1* gene (*Nf1*^+/−^ mice) (Elmadany et al., 2020b). We found that male, but not female, *Nf1*^+/−^ mice harbor microglia with impaired purinergic function, including reduced phagocytosis, microglia process extension and ATP-induced membrane currents *in vivo*.

These intriguing findings suggested that *NF1* mutation alters cell-intrinsic properties of microglia. In this study, to determine whether these *NF1* mutational effects were similarly observed in human male microglia in the setting of NF1, we leveraged an allelic series of CRISPR/Cas9-engineered male BJFF.6 hiPSCs harboring three different heterozygous *NF1* germline mutations found in patients with NF1. Here, we demonstrate that heterozygous *NF1* mutations in hiMGL cells recapitulate some, but not all, of the purinergic defects observed in their murine counterparts. These findings establish a foundation for future studies aimed at defining the cell-autonomous impact of germline mutations on human microglia function.

## RESULTS

### *NF1*-mutant hiMGL cells are phenotypically similar to isogenic control hiMGL cells

**Fig. 1.**
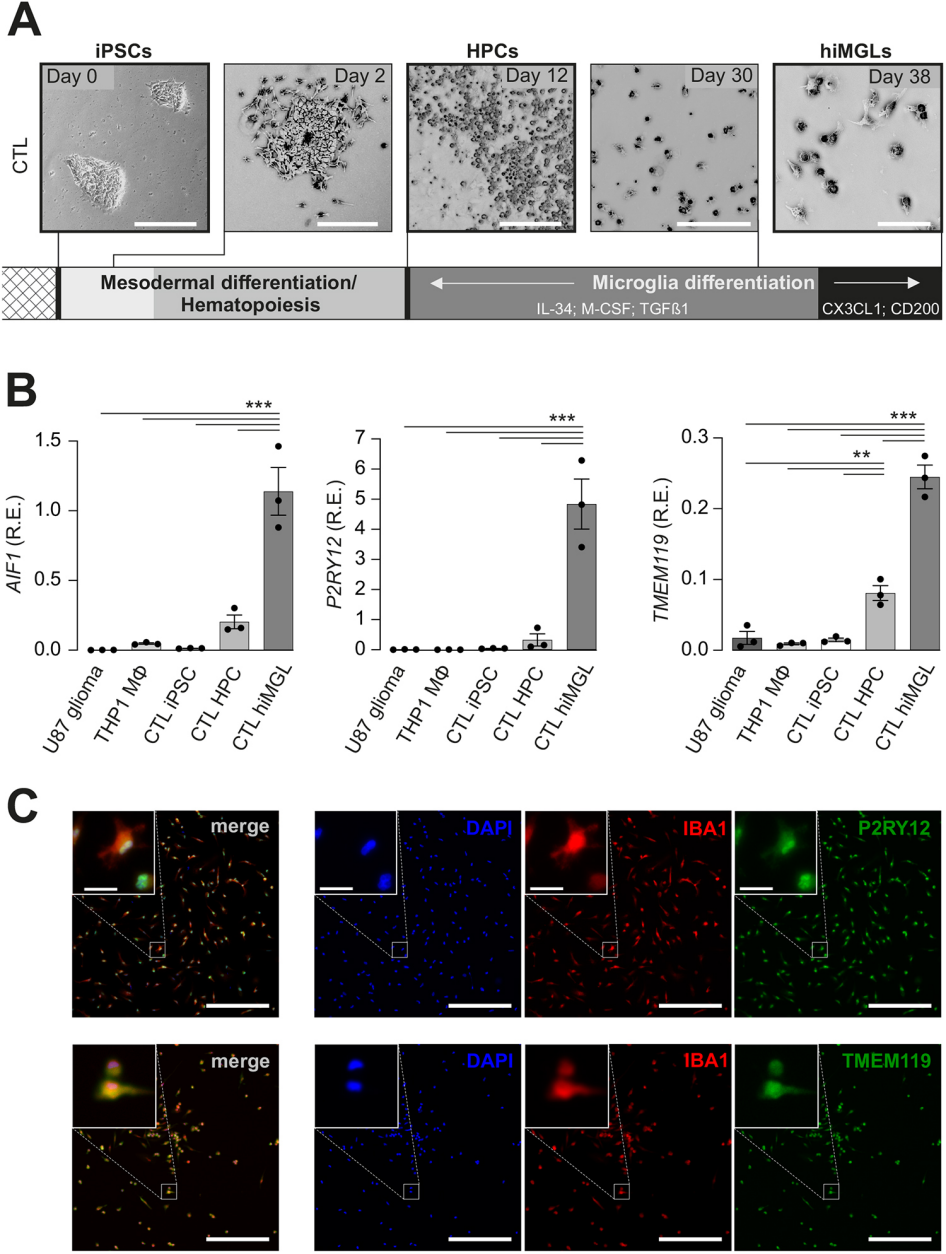
**hiMGL cell differentiation from control hiPSCs.** (A) Representative brightfield images of human induced microglia-like (hiMGL) cells differentiated from male BJFF.6 control (CTL) hiPSCs. Day 0: hiPSC cluster under regular hiPSC growth conditions. Mesodermal and hematopoietic differentiation was induced by the STEMdiff hematopoietic kit. Day 2: mesodermal differentiation. Day 12: hematopoietic progenitor cells (HPCs). Day 30: microglial differentiation using serum-free basal media supplemented with IL–34 (100 ng/ml), M–CSF (25 ng/ml) and TGFβ1 (50 ng/ml). Day 38: CX3CL1 (100 ng/ml) and CD200 (100 ng/ml) were also added for hiMGL cell maturation for the final three days *in vitro*. Scale bars: 200 μm (days 0, 2, 12 and 30); 50 μm (day 38). (B) Relative mRNA expression (R.E.) levels of the microglial markers *AIF1*, *P2RY12* and *TMEM119* were assessed in BJFF.6-derived control (CTL) hiPSCs, HPCs and hiMGL cells, as well as in THP-1-derived human macrophages (Mφ) and U87 human glioma cells by quantitative reverse transcription PCR (RT-PCR). Expression levels are shown relative to expression of the TATA box-binding protein (*TBP*) housekeeping gene (*n*=3). Results are represented as the mean±s.e.m. Data were analyzed by one-way ANOVA followed by Tukey's multiple comparisons test. **P< 0.01; ***P<0.001. (C) Immunohistochemical staining of BJFF.6-derived hiMGL cells with IBA1-, TMEM119- and P2RY2-specific antibodies. Nuclei were stained with DAPI. Merged images show the combined signal for DAPI, IBA1 and P2RY12 or TMEM119. Images are representative of three independent experiments. Scale bars: 200 μm (overview); 20 μm (inlay).

Using a previously published protocol (McQuade et al., 2018), we generated hiMGL cells from male control (CTL) BJFF.6 hiPSCs that had undergone CRISPR/Cas9 engineering without creating a *NF1* gene mutation (Fig. 1A). Briefly, hiPSCs were seeded at low density and hematopoiesis initiated using the STEMdiff hematopoietic kit. Floating hematopoietic progenitor cells (HPCs) were harvested and reseeded in Geltrex-coated six-well plates on day 12 post induction. Microglial differentiation was induced under serum-free conditions by the addition of human M-CSF (encoded by *CSF1*), human IL-34 and human TGF-β1 (encoded by *TGFB1*), prior to supplementation with human CD200 and human CX3CL1 for an additional 3 days. Throughout the course of microglial differentiation, cells developed ramifications and started to adhere to the wells by 38 days *in vitro* (Fig. 1A).

To validate their microglial identity, we assessed *AIF1*, *TMEM119* and *P2RY12* mRNA expression in the CTL lines at three different stages (hiPSCs, HPCs and hiMGL cells). For comparison, we analyzed the expression of these markers in human THP-1 macrophages (THP1 MΦ) and U87 human glioma cells (Fig. 1B). As expected for microglia-like cells, the expression of all three genes was increased in hiMGL cells compared to that in HPCs, whereas it was undetectable or the genes were expressed at very low levels in iPSCs, THP1 MΦ and U87 glioma cells. Immunocytochemical staining of these hiMGL cells confirmed the expression of IBA1 (encoded by *AIF1*), TMEM119 and P2RY12 at the protein level (Fig. 1C).

**Fig. 2.**
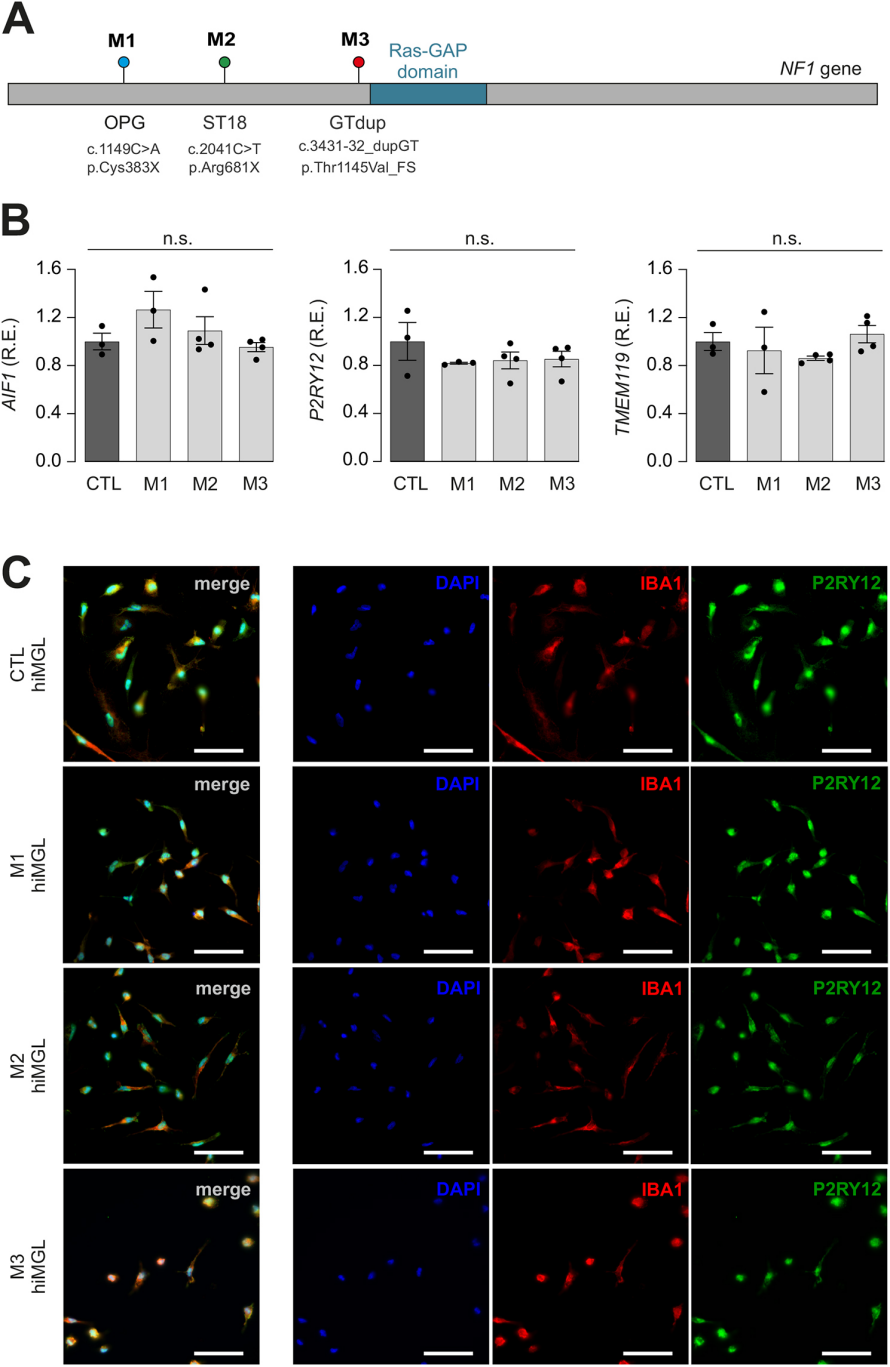
***NF1*-mutant hiPSCs differentiate into hiMGL cells.** (A) Schematic depicting the location of mutations engineered into the *NF1* gene locus by CRISPR/Cas9 editing of CTL hiPSCs, generating three different *NF1*-mutant hiPSC lines. Mutant 1 (M1), OPG c.1149C>A p.Cys383X; mutant 2 (M2), ST18 c.2041C>T p.Arg681X; mutant 3 (M3), dup_GT c.3431-32_dupGT p.Thr1145Val_FS. (B) Relative mRNA expression (R.E.) levels of the *AIF1*, *P2RY12* and *TMEM119* microglial markers were assessed in CTL and *NF1*-mutant hiMGL cells (M1-M3) by quantitative RT-PCR. Gene expression levels are shown relative to expression in CTL hiMGL cells. The housekeeping gene *TBP* was used for normalization (*n*=3). Results are represented as the mean±s.e.m. Data were analyzed using a one-way ANOVA followed by a Tukey's multiple comparisons test. No significant differences (n.s.) were found between CTL and M1-M3 hiMGL cells. (C) Immunohistochemical staining of CTL and *NF1*-mutant (M1-M3) hiMGL cells for IBA1 and P2RY12 expression. Nuclei were stained with DAPI. Merged images show the combined signal for DAPI, IBA1 and P2RY12. Images are representative of three independent experiments. Scale bars: 50 μm.

To generate hiMGL cells harboring *NF1* gene mutations, we used previously published hiPSC lines in which three different *NF1* gene mutations found in patients with NF1 were heterozygously introduced into BJFF.6 hiPSCs by CRISPR/Cas9 editing: c.1149C>A;p.Cys383X (mutant 1 or M1), c.2041C>T;p.Arg681X (mutant 2 or M2) and c.3431-32_dupGT;p.Thr1145Val_FS (mutant 3 or M3) (Fig. 2A) (Anastasaki et al., 2020). These mutations were chosen because they create different brain tumor phenotypes in genetically engineered *Nf1* mutant mice: despite the fact that all three mutations lead to premature termination (two stop codons and one frameshift mutation) and increased RAS activity, the Cys383X mutation results in lower optic glioma penetrance (Guo et al., 2019) than that for the Thr1145Val_FS mutation (J.C., unpublished), whereas the Arg681X mutation exhibits greater tumor proliferation (Toonen et al., 2016) than that for the Thr1145Val_FS mutation (J.C., unpublished). Moreover, we focused only on male hiPSC lines in light of our prior discovery of male-specific deficits in murine *Nf1*-mutant microglia.

Pluripotency and genomic integrity were confirmed (Fig. S1) prior to hiMGL cell differentiation. *AIF1*, *TMEM119* and *P2RY12* transcript expression (Fig. 2B), as well as IBA1, TMEM119 and P2RY12 protein expression (Fig. 2C; Fig. S2A), in *NF1-*mutant hiMGL cells was indistinguishable from those of isogenic CTL hiMGL cells. We also assessed *TREM2* expression in CTL and *NF1*-mutant hiMGL cell lines and found no differences in mRNA levels (Fig. S2B). In addition, we examined signaling pathway activity in *NF1*-mutant hiMGL cells. As our prior studies on *Nf1*-mutant murine microglia revealed that JNK, but not MAPK, activation was increased relative to that in their wild-type counterparts (Daginakatte et al., 2008), we analyzed JNK phosphorylation, and observed increased immunostaining for activated (phosphorylated) JNK in *NF1*-mutant hiMGL cells relative to that in their CTL hiMGL counterparts (Fig. S4).

### Transcriptome analysis reveals marked similarities between CTL and *NF1*-mutant hiMGL cells

**Fig. 3.**
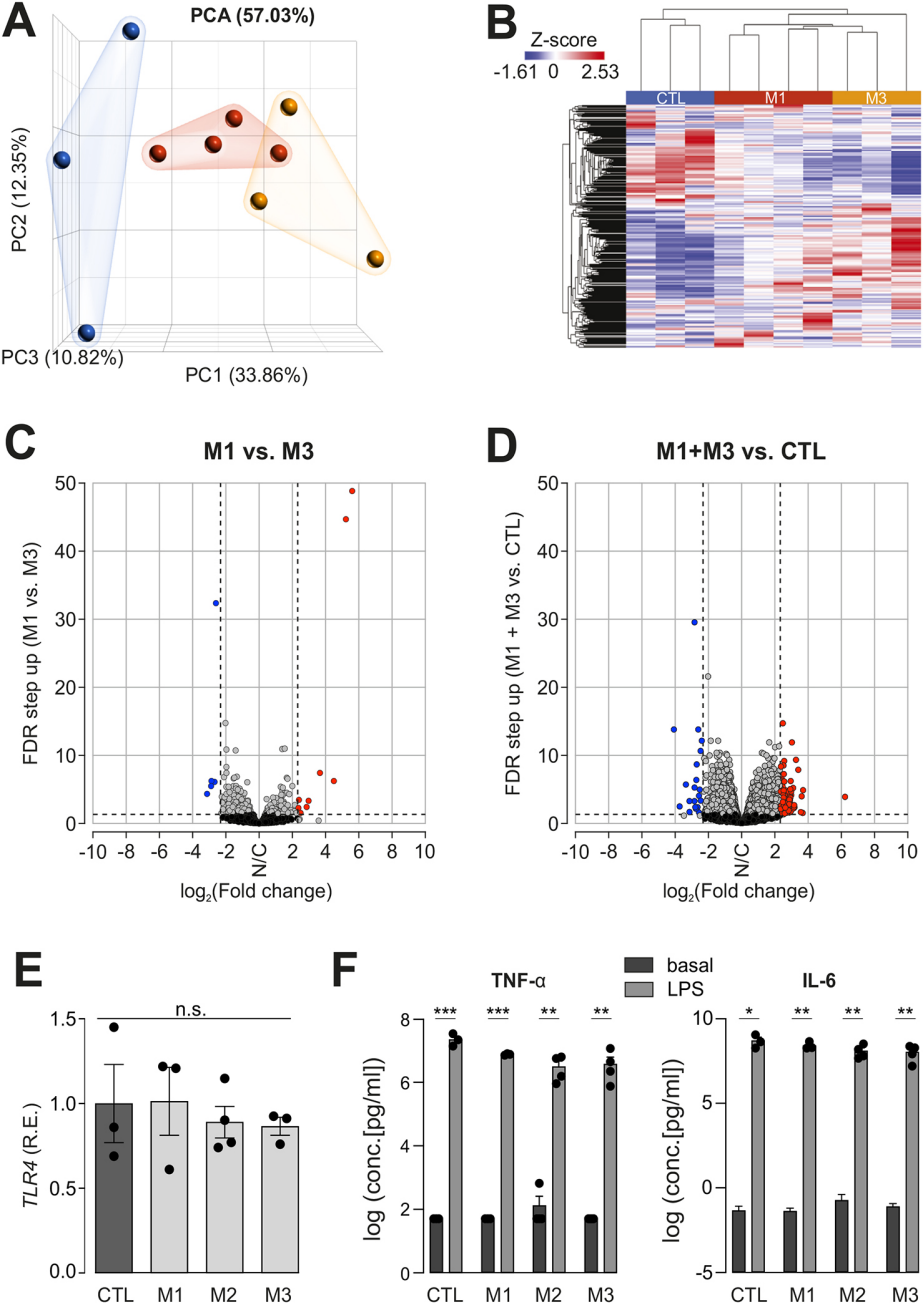
**RNA sequencing reveals few differences between *NF1*-mutant and CTL hiMGL cells, whereas the induced secretome remains unchanged.** (A) Principal component analysis (PCA) plot generated from RNA sequencing data (CTL, blue; M1, red; M3, yellow). (B) Heatmap showing unsupervised hierarchical clustering generated from RNA sequencing data for CTL, M1 and M3 hiMGL cells. (C,D) Volcano plot demonstrating genes differentially expressed between M1 versus M3 (C) or between M1 and M3 versus CTL (D) (FDR<0.05, fold change cutoff of −5 and 5). Grey dots indicate genes with no change (N/C) in expression, blue dots indicate genes with decreased expression and red dots indicate genes with increased expression. (E) Relative mRNA expression (R.E.) of Toll-like receptor 4 (*TLR4*) in CTL and *NF1*-mutant (M1-M3) hiMGL cells by quantitative RT-PCR. Gene expression levels are shown relative to expression in CTL hiMGL cells. The housekeeping gene *TBP* was used for normalization (*n*=3). Data were normalized to *TLR4* expression levels in CTL hiMGL cells. Results are represented as the mean±s.e.m. Data were analyzed by one-way ANOVA. (F) Multiplex immunoassay was used to detect TNF-α (left) and IL-6 (right) in supernatants from CTL and *NF1*-mutant (M1-M3) hiMGL cells in response to 1 μg/ml LPS stimulation for 24 h (n=3-4). Data between basal and LPS conditions were analyzed using two-tailed unpaired *t*-tests. Results are represented as the mean±s.e.m. Cytokine release under basal or LPS conditions was not significantly different between CTL and M1, M2 and M3 hiMGL cells (one-way ANOVA). The data showing the release of additional cytokines are included in Fig. S4. n.s., not significant; *P<0.05; **P<0.01; ***P<0.001.

To determine whether *NF1* mutation dramatically affected human microglia gene expression, we generated a minimum of three independent cultures from CTL and *NF1*-mutant hiMGL cells (M1 and M3) for bulk RNA sequencing analysis. Unfortunately, M2 *NF1*-mutant hiMGL cells could not be included in the analysis, as the resulting RNA failed quality control checks. Although *NF1*-mutant hiMGL cells were distinct from CTL hiMGL cells by both principal component analysis (Fig. 3A) and hierarchical clustering (heat map; Fig. 3B), there were few differences in the number of the differentially expressed genes between the two *NF1*-mutant hiMGL cell groups (0.047% of the genes expressed; Fig. 3C; Table S4) and only a modest number of differentially expressed genes between *NF1*-mutant and CTL hiMGL cells (0.044%; Fig. 3D; Table S3) using a cutoff of 5 for fold change in gene expression. Gene Ontology (KEGG) analysis and gene set enrichment analysis (GSEA) revealed no differentially represented pathways or biological functions between *NF1*-mutant hiMGL cells relative to those of their CTL counterparts (false discovery rate or FDR<0.25). Moreover, no significant differences in pathway or biological function enrichment were observed using fold change cutoffs of either 2 or 3 (Fig. S3).

### Secretome analysis reveals marked similarities between CTL and *NF1*-mutant hiMGL cells

Given the paucity of transcriptomal differences, we next analyzed the ability of *NF1*-mutant hiMGL cells to release inflammatory paracrine factors in response to lipopolysaccharide (LPS; 1 μg/ml) activation of Toll-like receptor 4 (TLR4). TLR4 activation was chosen as LPS is a commonly used agent to stimulate microglia function. *TLR4* RNA expression was similar in CTL and *NF1*-mutant hiMGL cells (Fig. 3E). The levels of secreted IL-6 and TNF-α (also known as TNFA) (Fig. 3F) as well as G-CSF (or CSF3), IL-1β (IL1B), GRO (CXCL1), IL-10, IP-10 (CXCL10), MCP-1 (CCL2), MCP-3 (CCL7), MIP-1β (CCL4), MIP-1α (CCL3) and CCL5 (RANTES) (Fig. S5) were similar in *NF1*-mutant and CTL hiMGL cells following 1 μg/ml LPS stimulation for 24 h.

### P2RY-dependent microglial membrane currents are similar in CTL and mutant hiMGL cells

As the RNA sequencing and secretome analyses did not reveal any significant differences, we next focused on purinergic abnormalities previously reported in male *Nf1*^+/−^ mouse microglia (Elmadany et al., 2020b). Microglia express a variety of purinergic receptors, including members of the metabotropic P2Y and ionotropic P2X receptor families. To determine whether *NF1*-mutant hiMGL cells express known P2Y and P2X receptors, we leveraged our RNA sequencing data (Fig. 3). Whereas M1 and M3 *NF1*-mutant hiMGL cells expressed similar levels of these receptors, we observed increased *P2RY4* RNA expression in the two *NF1*-mutants relative to that in their CTL counterparts (Fig. S7B).

First, we compared the membrane properties of microglia derived from CTL and *NF1*-mutant hiMGL cells. CTL hiMGL cells displayed microglia-characteristic membrane currents when repetitively clamped at potentials between −170 and 60 mV starting from a holding potential of −70 mV, similar to what we previously reported for mouse microglia (Elmadany et al., 2020b,a; Kettenmann et al., 2011; Logiacco et al., 2023; Boucsein et al., 2000) (Fig. S6A,B). These cells were characterized by a high input resistance and a small inwardly rectifying potassium conductance between −40 and −170 mV. Similar relationships were seen in *NF1*-mutant hiMGL cells (Fig. S6B). There were also no differences in reversal potentials (CTL: −21.0±2.3 mV, *n*=38; M1: −25.0±4.0 mV, *n*=17; M2: −23.7±3.9 mV, *n*=18; M3: −20.8±2.0 mV, *n*=20; indicated as mean±s.e.m; Fig. S6C), and the apparent membrane capacitances were indistinguishable from those of controls (CTL: 19.2±1.4 pF; M1: 20.7±2.2 pF; M2: 19.1±1.3 pF, *n*=18; M3: −16.6±1.6 pF; Fig. S6D).

**Fig. 4.**
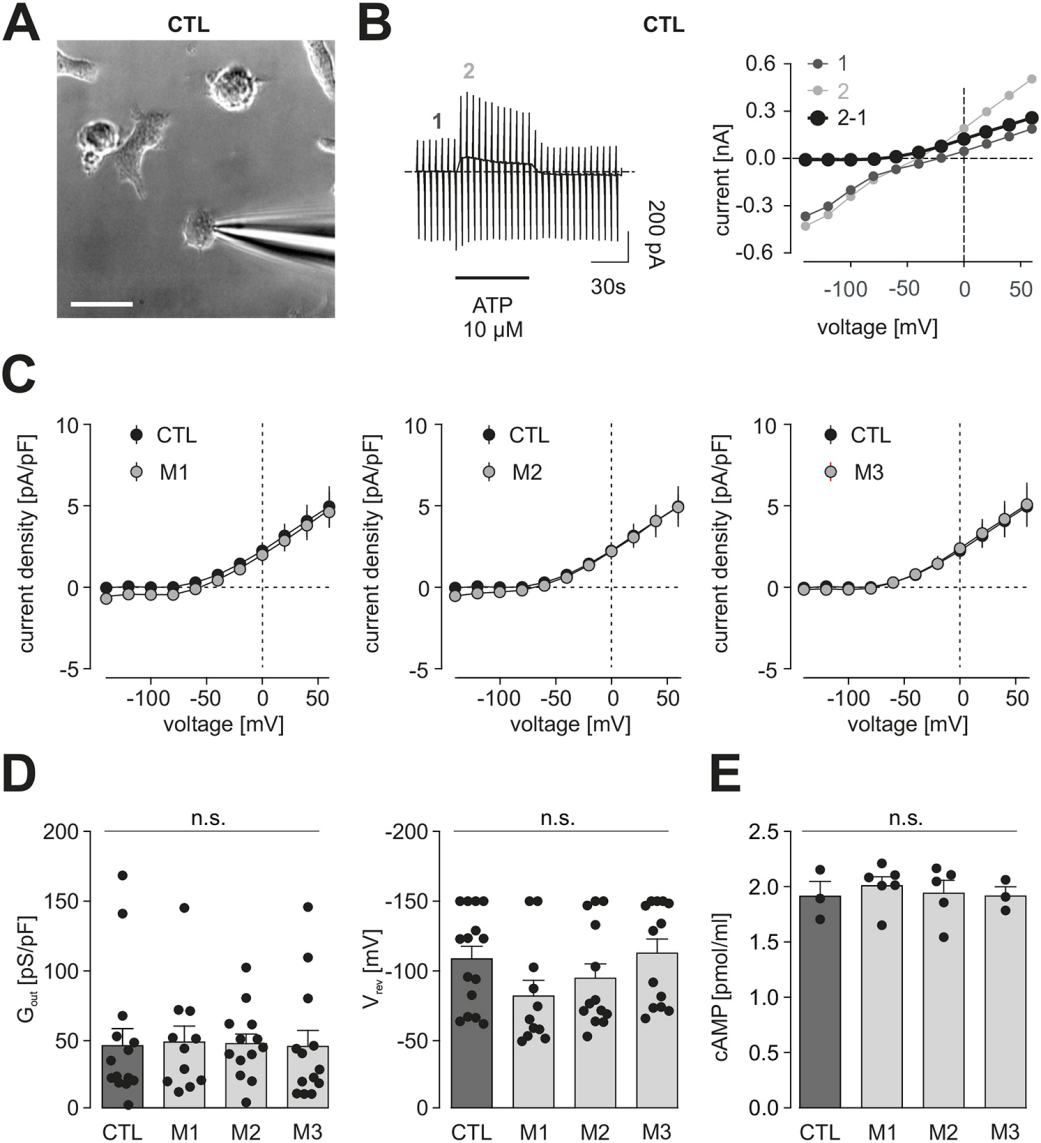
**Metabotropic purinergic responses are similar in CTL and *NF1*-mutant hiMGL cells.** (A) Representative transmission light microscopy image of CTL hiMGL cells with the patch pipette approaching the cell. Scale bar: 20 μm. (B) Representative patch-clamp experiments on CTL hiMGL cells. Left: membrane currents were recorded from a single CTL hiMGL cell. The membrane was repetitively clamped at potentials between −140 and 60 mV every 5 s from a holding potential of −20 mV. Application of 10 µM ATP is indicated by the bar. Note the typical microglial response to ATP with activation of currents in the outward direction. Right: current-voltage relationships were obtained from the current recordings on the left. Purinergic responses (black) were determined by subtraction of current-voltage relationships before (dark gray, 1) and during (light gray, 2) the first 15 s of ATP application. (C) Average current density to voltage relationships of ATP-induced metabotropic purinergic responses in microglia derived from CTL (black) and M1 (left, gray), M2 (middle, gray) or M3 (right, gray) *NF1*-mutant hiMGL cells. (D) Summary of the outward conductance (G_out_) between 20 mV and 60 mV (left) and the reversal potentials (V_rev_) (right) of ATP (10 µM)-evoked currents from CTL and *NF1*-mutant hiMGL cells. Data in C,D are presented as the mean±s.e.m. Statistical comparison in D was done by a one-way ANOVA. (E) cAMP levels of cell lysates from CTL and *NF1*-mutant hiMGL cells were measured by ELISA (*n*=3-6). Data in E are presented as the mean±s.e.m. For statistical analysis, one-way ANOVA was performed. n.s., not significant, *P*≥0.05. Number of experiments: CTL, *n*=15; M1, *n*=11; M2, *n*=13; M3, *n*=13.

To identify potential P2Y- and P2X-based deficits, we then examined P2RY12 responses using standard whole-cell patch-clamp techniques in the voltage-clamp configuration (Fig. 4), based on our previous report that murine male *Nf1*^+/−^ microglia had defects in P2RY12 activation (Elmadany et al., 2020b). Consistent with these previous studies, CTL hiMGL cells responded to 10 µM ATP with the induction of outwardly rectifying currents that reversed close to the equilibrium potential for potassium (Fig. 4A). We determined the specific conductance to compare these currents in CTL and *NF1*-mutant hiMGL cells between 20 and 60 mV (Fig. 4B). Both CTL and *NF1*-mutant hiMGL cells exhibited similar conductances (CTL: 44.8±12.5 pS/pF; *n*=15; M1: 47.4±11.7 pS/pF, *n*=11; M2: 46.3±7.2 pS/pF, *n*=13; M3: 44.2±11.8 pS/pF, *n*=13) (Fig. 4C,D). These findings contrast with what we observed in *Nf1*-mutant murine microglia (Elmadany et al., 2020b). In addition, unlike *Nf1*^+/−^ mouse microglia (Elmadany et al., 2020b), *NF1*-mutant hiMGL cells exhibited no differences in cyclic AMP (cAMP) levels relative to those of controls (Fig. 4E).

### P2X-dependent purinergic membrane currents are reduced in *NF1*-mutant hiMGL cells

**Fig. 5.**
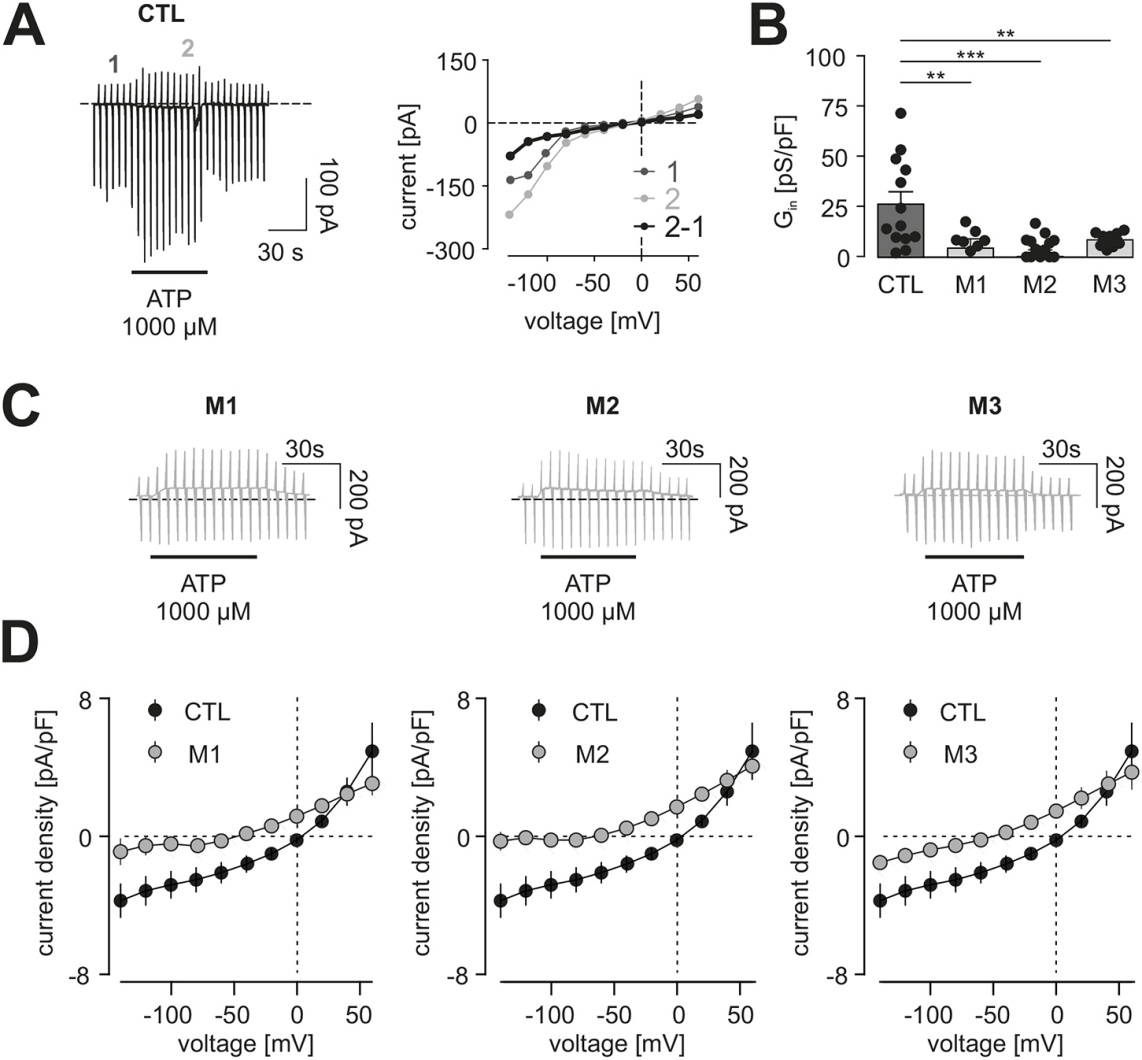
**Ionotropic purinergic responses are reduced in *NF1*-mutant hiMGL cells.** (A) Representative patch-clamp experiment. Left: membrane currents recorded from a single CTL hiMGL cell. From a holding potential of −20 mV, the membrane was repetitively clamped at potentials between −140 and 60 mV every 5 s. Application of 1000 µM ATP is indicated by the bar. Note the typical microglial response to ATP with activation of currents in inward and outward directions. Right: current-voltage relationships obtained from the recording on the left. The purinergic response (black) was determined by subtraction of current-voltage relationships before (dark gray, 1) and 15 s after (light gray, 2) the onset of ATP application. (B) Summary of the inward conductance (G_in_) of ATP (1000 µM)-evoked currents between −100 mV and −120 mV. Statistical comparison was performed using a one-way ANOVA followed by a Dunnett's multiple comparisons test. (C) Sample time courses of currents clamped at potentials as described in A of M1 (left), M2 (middle) and M3 (right) hiMGL cells during application of 1000 µM ATP as indicated by the bars. Note the absence of evoked inward currents. (D) Average current density to voltage relationships of ATP (1000 µM) responses in CTL hiMGL cells relative to those in the three *NF1*-mutant hiMGL cell lines. Data in B and D are presented as the mean±s.e.m. Number of experiments: CTL, *n*=13; M1, *n*=9; M2, *n*=14; M3, *n*=11. **P≤0.01; ***P≤0.001.

Given the lack of differences in P2Y function, we next analyzed P2X receptor-mediated responses. Microglia membrane currents were measured following the application of 1 mM ATP (Fig. 5), a concentration sufficient to activate P2RX4 and P2RX7, the most common P2X receptor isoforms in microglia (Butovsky et al., 2014; Ousingsawat et al., 2015). In CTL hiMGL cells, the application of 1 mM ATP slowly induced membrane currents within the application period (60 s), which were characterized by an inward and outward component and a reversal potential close to 0 mV, consistent with the characteristics of a non-selective cation channel (Fig. 5A). The specific conductance at −120 mV and –110 mV was 25.7±6.2 pS/pF (*n*=13) for CTL hiMGL cells (Fig. 5B). In contrast, 1 mM ATP did not evoke P2X-like ion currents in *NF1*-mutant hiMGL cells. Responses to 1 mM ATP were outwardly rectifying and had a reversal potential close to the Nernst potential for K^+^, appearing similar to the responses seen following 10 µM ATP stimulation (Fig. 5C). This result suggests that *NF1*-mutant hiMGL cells have defective P2X receptor expression or function. Although *NF1*-mutant hiMGL cells had similar levels of *P2RX4* and *P2RX7* RNA expression as that in CTL hiMGL cells (Fig. S7A), quantification of inward conductance as a measure of P2X-like currents revealed reduced conductances for M1 (3.5±4.0 pS/pF, *n*=9; *P*=0.0024), M2 (0.2±2.7 pS/pF, *n*=14; *P*<0.0001) and M3 (7.7±1.0 pS/pF, *n*=11; *P*=0.0095) *NF1*-mutant hiMGL cells relative to that of their CTL counterparts (Fig. 5D).

### Baseline motility is reduced in *NF1*-mutant hiMGL cells

**Fig. 6.**
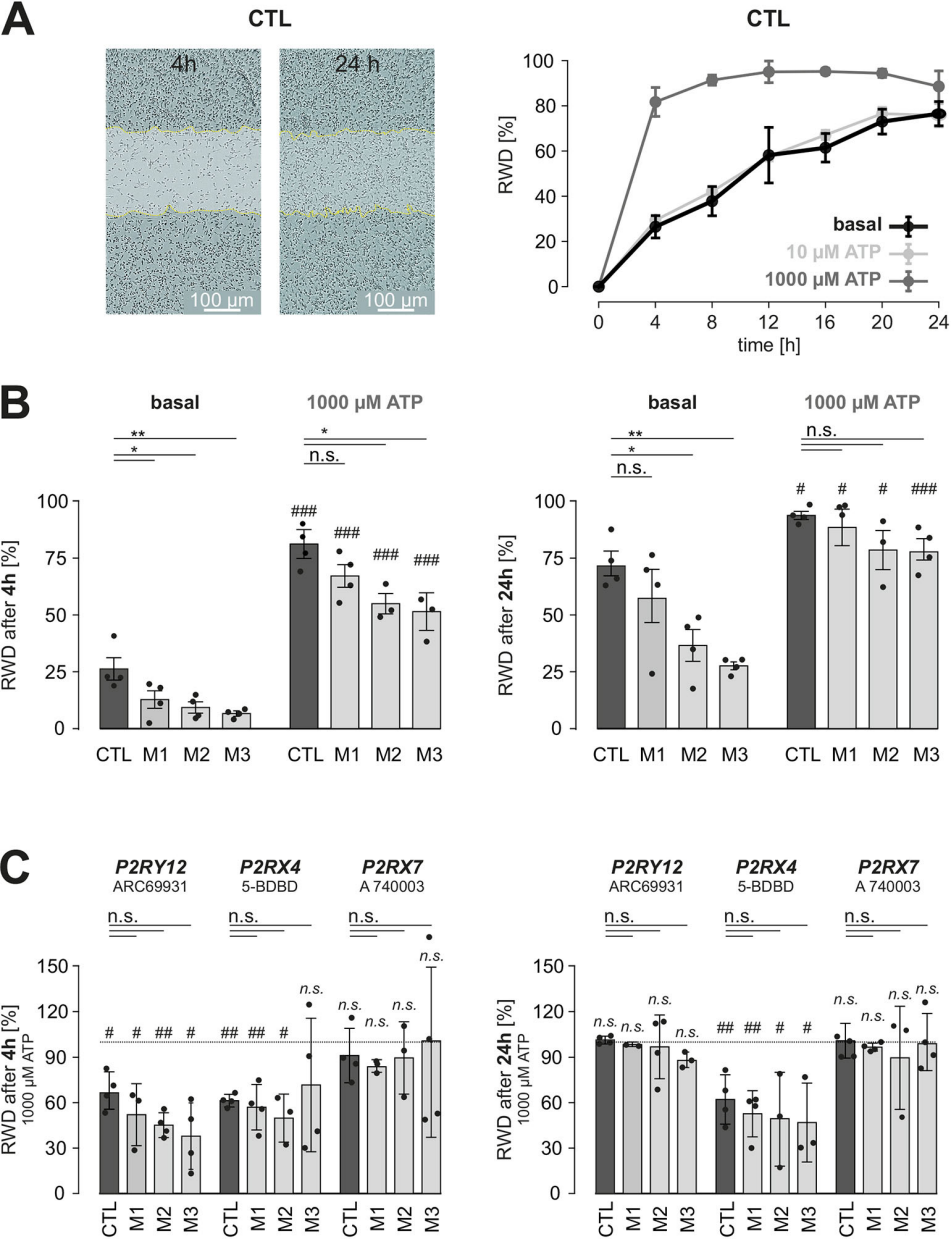
**Basal and ATP-induced motility in CTL and *NF1*-mutant hiMGL cells.** (A) Left: representative images from scratch wound experiments in CTL hiMGL cells analyzed at 4 h and 24 h after the initial wound. Color masks (white) and dotted lines (yellow) indicate the initial scratch wound area and border, respectively, at 0 h. Scale bars: 100 μm. Right: relative wound density (RWD) was assessed over the course of 24 h, comparing CTL hiMGL cell motility in the absence and presence of ATP (10 µM and 1000 µM). Data are represented as the mean±s.e.m. (B) RWD under control conditions and in the presence of 1000 µM ATP after 4 h (left) and 24 h (right), represented as mean±s.e.m. *n*=4 for CTL and *NF1*-mutants. (C) RWD in the presence of 1000 µM ATP and inhibitors of P2RY12 (10 µM ARC69931), P2RX4 (100 µM 5-BDBD) or P2RX7 (100 µM A 740003). Data are normalized to the RWD values for CTL or mutant hiMGL cells in the absence of inhibitors (1000 µM ATP only) and represented as the mean±s.e.m. For B,C, comparisons among the CTL, M1, M2 and M3 groups were performed using one-way ANOVA followed by Tukey's multiple comparisons test and significant differences are indicated with asterisks. Comparisons between basal and 1000 µM ATP conditions were performed using a two-tailed unpaired Student's *t*-test and significant differences are indicated with hashtags. n.s., not significant, *P*>0.05; *^/#^*P*<0.05; **^/##^*P*<0.01; ^###^*P*<0.001.

In order to assay other microglia functions, we tested hiMGL cell motility in a wound-healing scratch assay using an Incucyte Zoom live-cell imaging system with images automatically acquired every 4 h. The relative wound density (RWD) was calculated using Incucyte Zoom software for each time point recorded, where the RWD was plotted against time (Fig. 6A). Interestingly, treatment of CTL hiMGL cells with 10 µM ATP did not affect the number of cells in the scratched zone, indicating that ATP at that low concentration was not sufficient to alter cellular movements. In contrast, higher concentrations of ATP (1000 µM) led to faster wound healing, which was almost complete after 4 h (RWD: 81.7±45.7%), and therefore significantly faster than that observed under basal conditions without ATP (26.5±4.9%; *P*=0.0006).

We next investigated the impact of *NF1* mutation on hiMGL cell motility and found that *NF1*-mutant hiMGL cells had ∼30% reduced baseline motility relative to that of their CTL counterparts 4 h after generating the scratch (Fig. 6B, left). Exposure to 1000 µM ATP led to increased wound healing in CTL, M1, M2 and M3 hiMGL cells; however, the RWD observed in the M2 and M3 experiments was lower compared to that for CTL hiMGL cells. Likewise, after 24 h under basal conditions, only M1 hiMGL cells produced an increase in RWD comparable to that of CTL hiMGL cells, whereas the RWD for the M2 and M3 hiMGL cells was lower (Fig. 6B, right). Treatment of hiMGL cells with 1000 µM ATP led to an increase in RWD to ∼75-80% for both CTL and *NF1*-mutant hiMGL cells after 24 h. Taken together, these data indicate that the motility of hiMGL cells is reduced under basal conditions in an *NF1* mutation-dependent fashion.

Finally, we tested for the impact of the P2RY12, P2RX4 and P2RX7 purinergic receptors on ATP (1000 µM)-induced motility of CTL and *NF1*-mutant hiMGL cells and compared the RWD of each hiMGL cell line in the presence of 1000 µM ATP alone or with ARC-69931 (10 µM; P2RY12 inhibitor), 5-BDBD (100 µM; P2RX4 inhibitor) or A 740003 (100 µM; P2RX7 inhibitor) (Fig. 6C). Interestingly, P2RY12 inhibition led to a reduction in initial wound healing only, as the RWD for CTL and *NF1*-mutant hiMGL cells was decreased by ∼50% after 4 h, whereas inhibitor-induced reduction in RWD was no longer evident after 24 h. P2RX4 blockade led to a reduction in both initial and late ATP-induced wound healing, whereas P2RX7 inhibition did not affect ATP-induced motility. Notably, there were no differences in the impact of P2 receptor blockers on motility between CTL and *NF1*-mutant hiMGL cells, excluding the possibility that differences in P2 receptor activation account for the reduced motility observed in *NF1*-mutant hiMGL cells. This conclusion is further supported by our observation that *P2RY12* (Fig. 2B), *P2RX4* and *P2RX7* (Fig. S7A) mRNA expression levels are similar in CTL and *NF1*-mutant hiMGL cells.

To further investigate microglia motility, we chose to focus on Toll-like receptor 2 (*TLR2*) activation, which is known to increase random movement of murine microglia (Ifuku et al., 2016). *TLR2* RNA expression was similar between CTL and *NF1*-mutant hiMGL cells (Fig. S8A). hiMGL cells were incubated for 48 h with a known TLR2 ligand, PAM2CSK4 (PAM2; 100 ng/ml), prior to the scratch wound. Whereas CTL and *NF1*-mutant hiMGL cells exhibited similar increases in motility following PAM2 stimulation, *NF1*-mutant hiMGL cells had ∼30% reduced baseline motility relative to that of their CTL counterparts (Fig. S8B). In contrast, a related toll-like receptor, TLR8, was transcriptionally expressed at similar levels in all hiMGL cells, and microglia motility in response to the TLR8 activator ‘506’ did not differ between CTL and *NF1*-mutant hiMGL cells (Fig. S8C,D). These findings support a selective impairment in hiMGL cell function in the setting of *NF1* mutation.

### Induced phagocytosis is impaired in *NF1*-mutant hiMGL cells

**Fig. 7.**
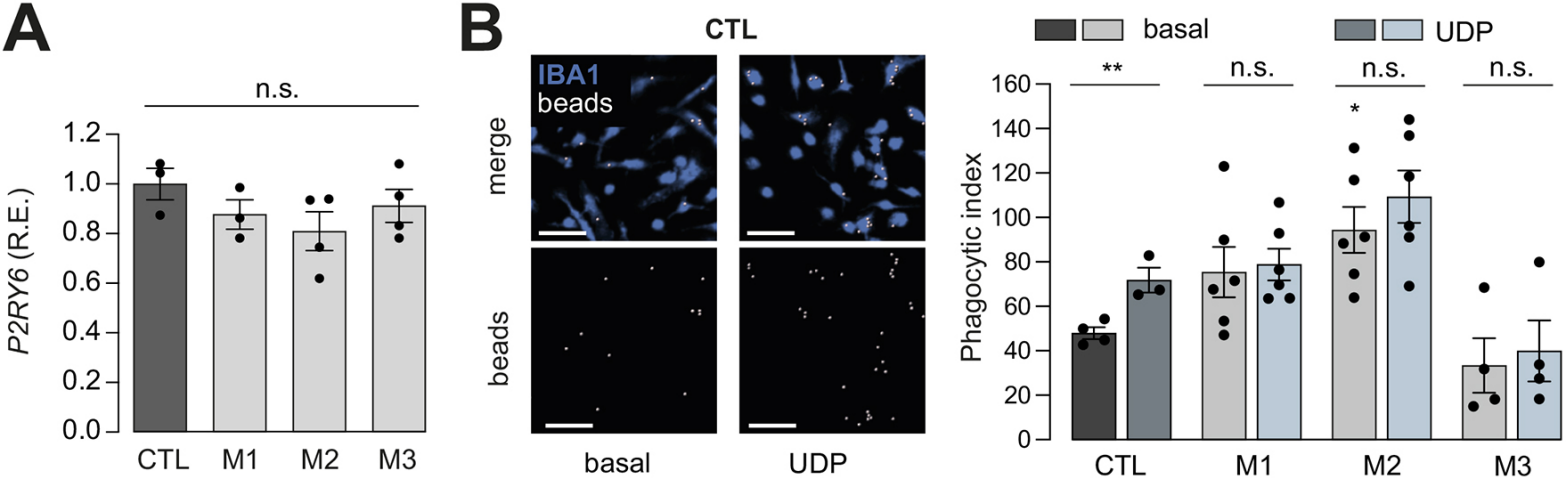
**Analysis of differences in phagocytic activity between CTL and *NF1*-mutant hiMGL cells.** (A) Relative *P2RY6* mRNA expression (R.E.) levels in CTL and *NF1*-mutant hiMGL cells by quantitative RT-PCR. Gene expression levels are shown relative to expression in CTL hiMGL cells. Data were normalized to *P2RY6* and *TBP* (housekeeping gene) expression in CTL hiMGL cells (*n*=3). Results are represented as the mean±s.e.m. Data were analyzed using a one-way ANOVA. (B) Phagocytic activity was assessed using fluorescent microbeads. CTL and *NF1*-mutant hiMGL cells were incubated for 1 h with the beads with or without the addition of 100 μM UDP. Left: representative images of CTL hiMGL cells at the end of the assay under basal and UDP conditions. IBA1 staining is indicated in blue and the beads are indicated in white. Top panels are an overlay of IBA1 (blue) and beads (white), whereas the lower panels show beads only. Control and UDP (100 µM) treatment conditions are shown in the left and right panels, respectively. Scale bars: 20 µm. Right: phagocytic activity presented as the phagocytic index, which is a measure of the percentage of cells exhibiting 0, 1, 2 or >3 engulfed beads. Results are represented as the mean±s.e.m. Comparisons among the CTL, M1, M2 and M3 groups were performed using one-way ANOVA followed by Tukey's multiple comparisons test. Comparisons between basal and UDP conditions were performed using a two-tailed unpaired Student's *t*-test. *n*=5 for CTL and *n*=3 for *NF1*-mutant hiMGL cells. Outliers were identified using Grubb's test and removed accordingly. n.s., not significant, *P*>0.05; **P<0.01.

In addition to their intrinsic electrophysiological properties, microglia interact with other cells in the brain through phagocytosis and motility. First, to examine the phagocytic activity of CTL and *NF1*-mutant hiMGL cells in response to P2RY6-dependent uridine diphosphate (UDP) stimulation (Koizumi et al., 2007), we measured *P2RY6* RNA expression in CTL and *NF1*-mutant hiMGL cells and found no differences between the two groups (Fig. 7A). Second, we quantified phagocytosis in at least three independent sets of CTL and *NF1*-mutant hiMGL cells following incubation for 45 min with yellow-green-fluorescent Fluoresbrite carboxylate polystyrene beads (Fig. 7B). Whereas M2 hiMGL cells showed a ∼50% increase in baseline phagocytic index relative to that of CTL cells, UDP treatment exclusively increased phagocytic activity in CTL hiMGL cells but not in *NF1*-mutant hiMGL cells.

To further study phagocytic activity in CTL and *NF1*-mutant hiMGL cells, we tested *TLR4* activation by LPS (1 µg/ml) (Fig. S9), as TLR4 is a major mediator of induced phagocytosis in response to LPS (Lee and Lee, 2002). In these experiments, LPS increased phagocytosis in CTL hiMGL cells, but failed to increase the phagocytic activity of the three *NF1*-mutant hiMGL cell lines. Taken together, these data suggest that *NF1*-mutant hiMGL cells sense some extracellular signals (UDP and LPS) but do not activate the intracellular pathways necessary to increase phagocytic capacity. Additional studies will be required to define the signaling defects conferred by *NF1* mutation in hiMGL cells.

## DISCUSSION

In this study, we evaluated the impact of three different germline *NF1* gene mutations found in patients with NF1 on human iPSC-derived microglia function. Although we found no major changes in the microglial transcriptome, basal membrane currents or cytokine secretion relative to those of their control counterparts, heterozygous *NF1* mutation (as seen in non-neoplastic cells from patients with NF1) caused alterations in P2X-dependent purinergic signaling, phagocytic activity and motility *in vitro* in hiMGL cells. These findings are in contrast with what we observed in mouse *Nf1*-mutant microglia, which exhibited no defects in P2X purinergic membrane currents (Elmadany et al., 2020b). Instead, male mouse *Nf1*-mutant microglia had impaired P2Y purinergic membrane currents and cAMP generation, whereas their *NF1*-mutant hiMGL cell counterparts exhibited P2Y-mediated currents and cAMP levels indistinguishable from those of CTL hiMGL cells. The observations reported herein highlight the differences in the effects of germline *NF1* mutations on mouse and human microglia function.

Although the exact etiologies for these differences remain unelucidated, several possibilities exist. First, the prior experiments on *Nf1^+/−^* mouse microglia were performed on brain slices *in situ*, in which microglia can respond to local signals from other *Nf1*-mutant cell types (e.g. neurons). In this regard, microglia are very sensitive to environmental changes and can alter their expression profile within hours following removal from the brain (Gosselin et al., 2017). For instance, microglia markers, such as *P2RY12*, *CX3CR1* and *CD11B* (or *ITGAM*), are downregulated during brain microglia isolation (Mizee et al., 2017). In addition, some transcripts expressed by microglia *in situ* using laser capture microdissection are not found following isolation by fluorescence-activated cell sorting (Solga et al., 2015b). Moreover, microglia maintained *in vitro* can express cytokine transcripts not expressed *in vivo* (Hurley et al., 1999), such that enzymatic dissociation can induce transcriptional bias not reflective of their *in vivo* state (Mattei et al., 2020).

Second, it is conceivable that the hiMGL cells are not fully mature as a result of *in vitro* differentiation and lack some of the functional properties seen in their adult (12- to 16-week-old) mouse counterparts. As these microglia were induced through the differentiation of HPCs using defined soluble factors, they did not derive from the yolk sac and mature in the context of a developing brain. In support of this notion, human iPSC-derived microglia adopt a more *in vivo*-like expression pattern following transplantation into the mouse brain (Xu et al., 2020).

Third, there could be species-specific differences between human and mouse microglia relevant to inflammatory responses, as well as factors involved in brain development (Galatro et al., 2017; Gerrits et al., 2020; Jurga et al., 2020; Smith and Dragunow, 2014). Other studies found greater heterogeneity in human microglia populations *ex vivo* compared to murine microglia. These differences likely relate to the shorter life span and more controlled living environment of laboratory animals (Abels et al., 2021; Galatro et al., 2017; Geirsdottir et al., 2019; Jurga et al., 2020). In a study analyzing glioma-associated microglia, differential expression of species-specific signaling molecules that mediate microglia-glioma interactions were noted (Landry et al., 2012). As such, the glioma growth-promoting cytokine, CCL18, is expressed by human microglia, but not by murine microglia. Additionally, murine microglia produce a significant amount of nitric oxide upon stimulation with LPS, whereas human microglia do not (Landry et al., 2012). Moreover, murine and human microglia have different and sometimes even opposite reactions to drug treatments. For example, valproic acid induces cell death through caspase-3 activation in rodent, but not human, microglia (Smith and Dragunow, 2014). Valproic acid has also been shown to increase phagocytic activity of rodent microglia, whereas it inhibits phagocytosis in human microglia (Gibbons et al., 2011; Smith et al., 2010). Lastly, murine microglia have higher proliferation capacities *in vitro* than human microglia do (Smith and Dragunow, 2014).

Taken together, the results described in this report reveal defects in human *NF1*-mutant microglia, as well as differences and similarities in function between hiMGL cells and mouse *Nf1*-mutant microglia. Future studies will be required to determine whether these similarities and differences reflect species-specific effects or the impact of the brain environment on microglia function *in vivo*.

## MATERIALS AND METHODS

### hiPSC quality control

The original CTL BJFF.6 hiPSCs were generated from commercially available human foreskin fibroblasts (Stemgent, Reprocell, MD, USA). All NF1-mutant hiPSC lines were derived from the CTL BJFF.6 hiPSC line as previously described (Anastasaki et al., 2020). All hiPSC lines used in this project underwent extensive quality control before being used for microglial differentiation. Sterility for microorganisms was assessed first by antibiotic-free culture for a week. Cells were checked daily under the microscope and showed no signs of microbiological contamination. Next, cells were tested for mycoplasma using the Promokine Mycoplasma Test Kit I/C (Minerva Biolabs, Berlin, Germany). All cell lines used tested negative for contamination with mycoplasma, bacteria, yeast or fungi. Viability appeared normal, and growth to confluence was typical of that of hiPSCs. Pluripotency of hiPSCs was confirmed through immunocytochemistry for pluripotency markers (OCT3/4, NANOG, TRA-1-60 and SSEA-4) (Fig. S1). hiPSCs were karyotyped using the iScan machine (Illumina, CA, USA) and the Illumina platform OMNI-Express-8v1.6 Chip (coverage of 958,497 markers spanning the whole human genome). The analysis was performed using the Karyostudio 1.3 software (Illumina) based on the information of the GRCh36/hg18 dataset. All cell lines had an inconspicuous karyotype showing no larger areas of deletions or insertions (Fig. S1). All alterations that were detected were well below the 5 megabase pair (Mbp) threshold detectable by regular G-banding. A 1.7 Mbp gain (chromosome 20) in the CTL hiPSC line was the largest gain found. A similar gain was found in all *NF1*-mutant lines (Fig. S1). Cell line identity (short tandem repeat analysis) was performed using the GenePrint 10 System (Promega). The analysis confirmed that all hiPSC lines were derived from the same donor.

### hiPSC culture

hiPSCs were grown on Geltrex-coated vessels (0.12-0.18 mg/ml, Life Technologies, Grand Island, NY, USA), incubated in StemMACS iPS-Brew XF medium (Miltenyi Biotec, Bergisch Gladbach, Germany), passaged weekly using StemPro Accutase (Life Technologies), and supplemented with 0.5 μM thiazovivin (STEMCELL Technologies, Vancouver, BC, Canada) for the first 24 h after passaging. hiPSCs were frozen in Bambanker (GC Lymphotec, Tokyo, Japan) for long-term liquid nitrogen storage. hiPSCs used for microglial differentiation were kept in culture for a maximum of 3 weeks. See Table S5 for details of materials used.

### Microglial differentiation

hiMGL cells were generated from hiPSCs as previously described (McQuade et al., 2018) with minor modifications. On day −1, about 60-100 hiPSC clusters were seeded onto a Geltrex-coated six-well plate (Falcon, Corning, Glendale, AZ, USA) (Fig. 1A) with fresh StemMACS medium. On day 0 (induction), wells containing approximately 50 clusters at a diameter of 100-200 μm each were used for hematopoietic differentiation. HPCs were generated using the STEMdiff hematopoietic kit (STEMCELL Technologies). On day 0, the medium was changed to Medium A (included in the STEMdiff kit) for the initial 3 days of the protocol. On day 3, Medium A was exchanged for Medium B (included in the STEMdiff kit) and cells were cultured for an additional 9 days. The first HPCs arose on day 7. On day 12, HPCs, which have been reported to be CD43^+^ (McQuade et al., 2018), were harvested and replated (1.2×10^5^ cells per six-well plate) into DMEM/F12 (no Phenol Red) (Life Technologies)-based serum-free microglia differentiation medium with B27 (2×) (Life Technologies), insulin-transferrin-selenite (2×) (Life Technologies), N-2 (0.5×) (Life Technologies), GlutaMAX (1×) (Life Technologies), MEM non-essential amino acids solution (1×) (Life Technologies), insulin (5 μg/ml; PromoCell, Heidelberg, Germany) and α-thioglycerol (400 μM; Merck, Darmstadt, Germany). The microglia differentiation medium was supplemented with IL-34 (100 ng/ml; Peprotech, Cranbury, NJ, USA), TGF-β1 (50 ng/ml; Peprotech,) and M-CSF (25 ng/ml; Peprotech). Medium with fresh supplements (IL-34, TGF-β1 and M-CSF) was added every other day. On days 12 and 25 after replating the HPCs, excess medium was carefully removed, leaving 3 ml/well behind. On day 25 and again on day 27 post replating, CD200 (100 ng/ml; Novoprotein America, Fremont, CA, USA) and fractalkine (100 ng/ml; Peprotech) were additionally added to the medium to support microglial maturation. hiMGL cells were harvested between days 40 and 43 post induction (days 28 and 31 post replating, respectively).

### Immunocytochemistry

hiMGL cells were seeded onto 12 mm glass cover­slips in 24-well plates (Sarstedt, Nümbrecht, Germany). Cells were fixed, washed with PBS, and treated with blocking solution [5% normal donkey serum (Merck) and 0.1% Triton X-100 (Roth, Karlsruhe, Germany)] for 1 h. Cells were incubated with primary antibodies overnight at 4°C (Table S1). Subsequently, they were washed three times with PBS and incubated with an appropriate secondary antibody for 2 h at room temperature (Table S1). Nuclei were counterstained with 4′,6-diamidino-2-phenylindole (DAPI) solution (Merck) for 20 min. Cells were washed three times with PBS and coverslips were attached onto glass slides using one drop of Aqua-Poly/Mount (Polysciences Europe, Hirschberg an der Bergstraße, Germany). Images were acquired using a Leica DMi8 Thunder Imager 3D microscope with a Leica DMC 2900 camera or a Leica DMi8 microscope with a Leica DFC450 C camera (Leica Microsystems, Wetzlar, Germany).

### Quantitative reverse transcription PCR

For gene expression analysis, cells were harvested and RNA was isolated with the ReliaPrep RNA tissue kit (Promega, Fitchburg, USA) according to the manufacturer's instructions. RNA purity and concentration were measured using a NanoDrop 8000 spectrometer (Thermo Fisher Scientific, Schwerte, Germany) and RNA was reverse transcribed into cDNA using the PrimeScript RT reagent kit (Takara Bio, Kusatsu, Japan). Duplicate quantitative PCR was performed using a 7500 Fast Real-Time thermocycler (Applied Biosystems, Carlsbad, USA) with the SYBR Green Master Mix (Life Technologies). TATA box-binding protein (*TBP*) was used as the housekeeping gene (Table S2). Cycle threshold (Ct) values were recorded for each sample for the target gene and the housekeeping gene and ΔCt values were calculated. Expression is shown relative to the expression of the housekeeping gene in CTL hiMGL cells.

### Scratch wound assay

Cell motility was assessed using the IncuCyte cell migration kit (Sartorius, Göttingen, Germany) and an Incucyte Zoom live cell analysis system (Sartorius). 100,000 hiMGL cells were seeded onto a 96-well ImageLock plate (Sartorius) and incubated for 4 h in serum-free microglia maturation medium supplemented with IL-34, TGF-β1, M-CSF, CD200 and fractalkine. Scratches were then made in all wells simultaneously using the IncuCyte cell migration kit (Sartorius). Inhibitors and agonists were added according to the respective experimental conditions: 5-BDBD (100 μM; P2RX4 inhibitor), ARC-69931 (10 μM; P2RY12 inhibitor), A 740003 (100 μM; P2RX7 inhibitor), ATP (10 μM/1000 μM), PAM2CSK4 (100 ng/ml; TLR2 agonist) and 506 (100 ng/ml; TLR8 agonist) (Table S5). Plates were then placed into the IncuCyte Zoom live cell analysis system, where hourly images were taken for 48 h at 4× magnification. Data were acquired using the scratch wound analysis tool from the IncuCyte Zoom software (Sartorius) and presented as RWD as a percentage.

For each experiment, a new processing definition was set by adjusting segmentation bias and setting a minimal object area of 500 µm^2^. Confluency was measured and expressed as the percentage of scratch wound surface covered by the migrating cells. Confluency was normalized to 0% confluency at t=0.

### Electrophysiological recordings

An Axioskop FS2plus (Zeiss, Oberkochen, Germany), a conventional patch-clamp amplifier (EPC9, HEKA Elektronik, Lambrecht, Germany) and TIDA 5.23 software (HEKA Elektronik, Lambrecht, Germany) were used. Patch pipettes were pulled from borosilicate glass with resistances of 3–8 MΩ. The intracellular solution used contained 130 mM KCl, 2 mM MgCl_2_, 0.5 mM CaCl_2_, 2 mM Na-ATP, 5 mM EGTA and 10 mM HEPES and had an osmolarity of 310-320 mOsm/l adjusted to a pH of 7.3 with KOH. The extracellular solution contained 140 mM NaCl, 5 mM KCl, 1 mM MgCl_2_, 2 mM CaCl_2_, 10 mM HEPES and 10 mM D-glucose, with pH 7.4 and osmolarity 270-290 mOsm/l. Experiments with series resistances less than 100 MΩ were used for data analysis. All experiments were performed in the voltage-clamp configuration. To obtain current-voltage curves during continuous recordings, the membrane was clamped every for 5 s from a holding potential of either −70 mV (membrane properties; Fig. S6) or −20 mV (ATP responses; Figs 4 and 5) to a series of 50 ms depolarizing and hyperpolarizing voltages ranging from −140 to 60 mV with 10 or 20 mV increments. Membrane currents were averaged for quantification between 30 and 45 ms after clamping the membrane to a given value from the resting potential. Membrane capacitance was quantified based on an exponential fit of the current decay in response to a −10 mV test pulse. The same pulse was used to quantify series resistance from the peak amplitude of the membrane capacitance currents. Comparisons of membrane currents between different groups were normalized to the membrane capacitance.

### *In vitro* phagocytosis assay

The phagocytosis assays were performed as previously described (Pannell et al., 2016) with some adaptations. hiMGL cells were seeded onto glass coverslips in 24-well plates (2.5×10^5^ cells/well), incubated in serum-free microglia maturation medium supplemented with IL-34, TGF-β1, M-CSF, CD200 and fractalkine for 4 h, and were stimulated with or without 1 μg/ml LPS (Merck). Yellow-green-fluorescent Fluoresbrite carboxylated microbeads (3.00 µm diameter, Polysciences Europe) were pre-opsonized with fetal calf serum (FCS, Life Technologies) by shaking at 1000 rpm for 30 min at room temperature. After centrifugation for 2 min at 900 ***g***, the supernatant was discarded and microbeads were washed and resuspended in HBSS (Life Technologies). hiMGL cells were incubated with the microbead suspension (approximately 4.8×10^6^ microbeads/well) containing either no stimulant (basal), UDP (100 µM; Abcam, ab120383) or LPS (1 μg/ml) for 45 min. Cells were then washed with PBS (Life Technologies). Coverslips were fixed with 4% paraformaldehyde, washed and blocked in TBS containing 5% donkey serum and 0.1% Triton X-100 for 1 h. Cells were next stained with anti-IBA1 antibody (Table S1) in TBS containing 5% donkey serum overnight. After washing, cells were incubated with a secondary Alexa Fluor 647-conjugated antibody (Table S1) and DAPI before mounting with Aqua-Poly/Mount. Three z-stack images per coverslip were acquired using a Leica TCS SPE confocal laser microscope (Leica Microsystems). Images were 3D-rendered using Imaris software (Imaris 9, Oxford Instruments, Abingdon, UK). IBA1 surfaces and spots for DAPI and microbeads were created. To represent the hiMGL cell number per image, DAPI spots within IBA1 surfaces were assessed using the ‘split into object’ function. To represent the phagocytosed beads, the numbers of beads within IBA1 surfaces were counted. Using in-house developed Python software, cells were grouped as having 0, 0 to 1, 1 to 2, 2 to 3, or >3 beads per cell. The percentage of cells in each group was multiplied by the corresponding grade of phagocytosis: 0, grade 1; 0 to 1, grade 2; 1 to 2, grade 3; 2 to 3, grade 4; and >3, grade 5. The sum of the products in each group was then defined as the phagocytosis index.

### cAMP ELISA

cAMP levels in hiMGL cells were determined using the cAMP ELISA kit (Cayman Chemical, Ann Arbor, MI, USA) according to the manufacturer's instructions. Briefly, hiMGL cells were resuspended in 100 μl of 0.1 M HCl and centrifuged for 10 min at 1000 ***g***. Supernatants were neutralized with ELISA buffer at a ratio of 1:2. Next, sample acetylation was performed by adding 60 μl of 4 M KOH and 15 μl acetic anhydride, vortexing for 15 s, followed by the addition of 15 μl of 4 M KOH. Samples and standards were pipetted into the ELISA plates and incubated for 18 h at 4°C before the plate was developed with Ellmann's reagent (included in the cAMP ELISA kit). Fluorescence was measured at 420 nm using a Tecan Infinite M200 plate reader (Tecan Group, Männedorf, Switzerland)

### Multiplex ELISA

The cell supernatant (25 μl) was analyzed using the MagPix System (Luminex/Merck). The MILLIPLEX MAP human cytokine/chemokine/growth factor panel A – immunology multiplex assay (HCYTA-60K, Merck) was used according to the manufacturer's recommendations. The following analytes were included in the analysis: GM-CSF, GRO, IL-1β, IL-6, IL-10, IP-10, MCP-1, MCP-3, MIP-1α, MIP-1β, RANTES and TNF-α. Xponent (Luminex) and Milliplex Analyst (Merck) software was used for data acquisition. Background intensity (pure medium) was subtracted from the mean fluorescent intensity. Concentration (pg/ml) was determined using an 8-point calibration curve including the matrix (cell culture medium). The standards were measured twice. Samples were measured only once. To determine the instrumental variance, pooled samples were used as quality control.

### RNA sequencing and analysis

RNA was extracted from four independently generated hiMGL cell cultures for each genotype (CTL, M1 and M3) and subjected to sequencing on an Illumina HiSeq platform. RNA sequencing analysis was generated using Partek Flow software, v10.0 (https://www.partek.com/partek-flow/). RNA sequencing reads were aligned to the hg38 Ensembl release 105 assembly with STAR v2.7.8a (Dobin et al., 2013). Gene counts and isoform expression were derived from the Ensembl output. Sequencing performance was assessed for the total number of aligned reads, total number of uniquely aligned reads and features detected. Normalization size factors were calculated for all gene counts by median ratio. Differential genetic analysis was then performed using DESeq2 (Love et al., 2014) to analyze for differences between conditions. Results were filtered for only those genes with FDR≤0.05 and fold changes more than ±5. Additional analyses were performed with cutoff criteria of >2 or >3 fold change. These data have been deposited in the Gene Expression Omnibus (accession number GSE203647).

### Statistics

Data are expressed as mean±s.e.m. A combination of one-way ANOVA followed by Dunnett's post hoc test or Tukey's multiple comparisons test was employed to compare data between more than two experimental groups. Two-tailed unpaired Student's *t*-test was used to compare data between two experimental conditions. IGOR Pro 6.34 (WaveMetrics, Lake Oswego, OR, USA; https://www.wavemetrics.com/) or GraphPad Prism 9 (GraphPad Software, San Diego, CA, USA) was used for statistical analysis. Information about the statistical tests and number of experiments is specified in the figure legends. Significance levels are given as: n.s., not significant, *P*>0.05; *^/#^*P*<0.05; **^/##^*P*<0.01; ***^/###^*P*<0.001.

## Supplementary Material

10.1242/dmm.049861_sup1 Supplementary information
