# Supplementary material for: Neurofibromin 1 mutations impair the function of human induced pluripotent stem cell-derived microglia
Source: Dis Model Mech. 2023 Dec 11;16(12):dmm049861. doi: 10.1242/dmm.049861 (PMC10740172; doi:10.1242/dmm.049861)
Supplement: Supplementary information [file dmm-16-049861-s1.pdf]

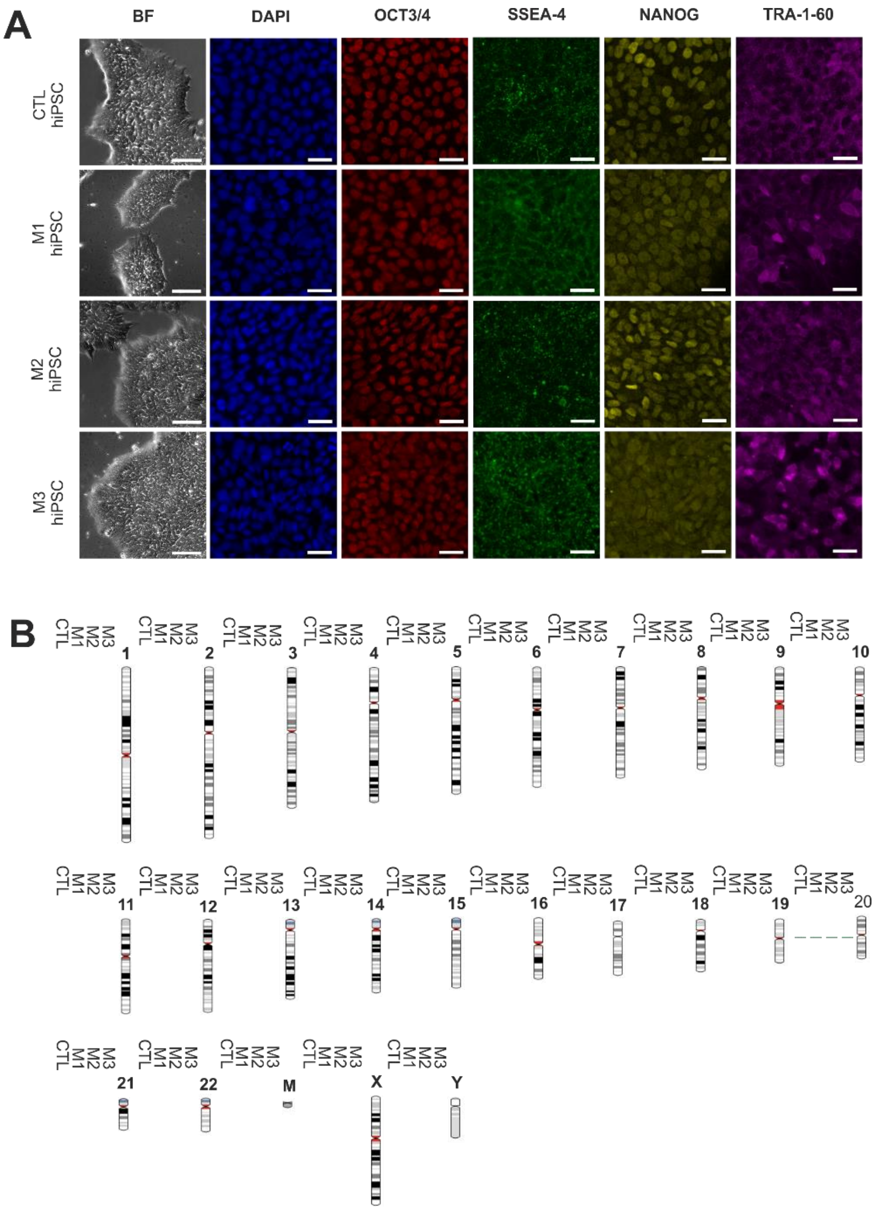

Fig. S1. Pluripotency immunocytochemistry and single nucleotide polymorphism (SNP)-karyotyping of CTL and *NF1*-mutant hiPSCs

A Brightfield (BF) images and immunocytochemical analysis of CTL and *NF1*-mutant hiPSC cells for OCT3/4, SSEA-4, NANOG and TRA-160 pluripotency markers. Scale bars, 100 µm (BF) and 30 µm.

B Virtual karyotype generated through SNP-karyotyping for all hiPSC lines. Areas of gain are marked in green, areas of loss in red, and loss of heterozygosity in gray.

Disease Models & Mechanisms • Supplementary information

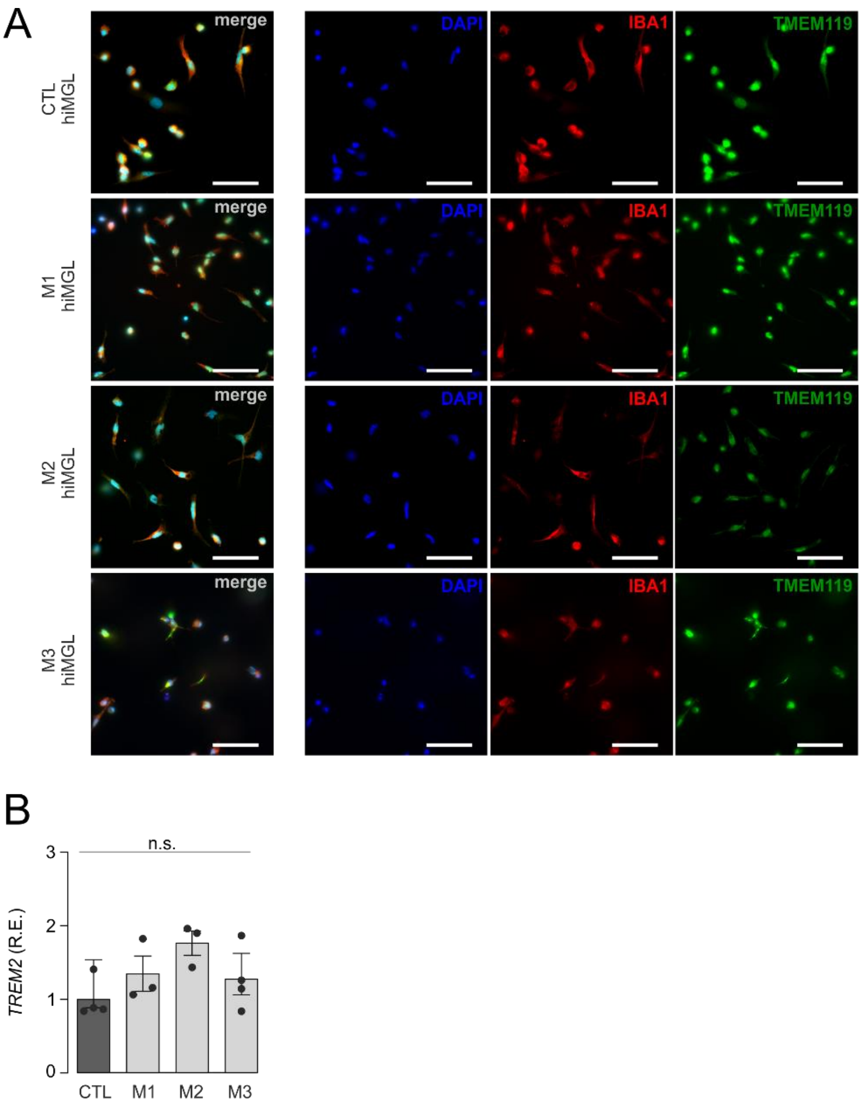

Fig. S2. Immunohistochemical staining and TREM2 expression in CTL and *NF1*-mutant hiMGL cells

A CTL and *NF1*-mutant hiMGL cells labelled with DAPI are immunopositive for IBA1 and TMEM119 expression. Merged images show the combined signal for DAPI, IBA1 and TMEM119. Scale bars, 50 µm.

B Relative mRNA expression levels of the *TREM2* microglia marker were assessed in CTL and *NF1*-mutant hiMGL cells by quantitative RT-qPCR. Relative expression (R.E.) was normalized relative to the TATA box binding protein (*TBP*) housekeeping gene (n=3). Results are represented as the mean ± SEM. Data were analyzed by one-way ANOVA followed by Tukey's multiple comparisons test.

Disease Models & Mechanisms • Supplementary information

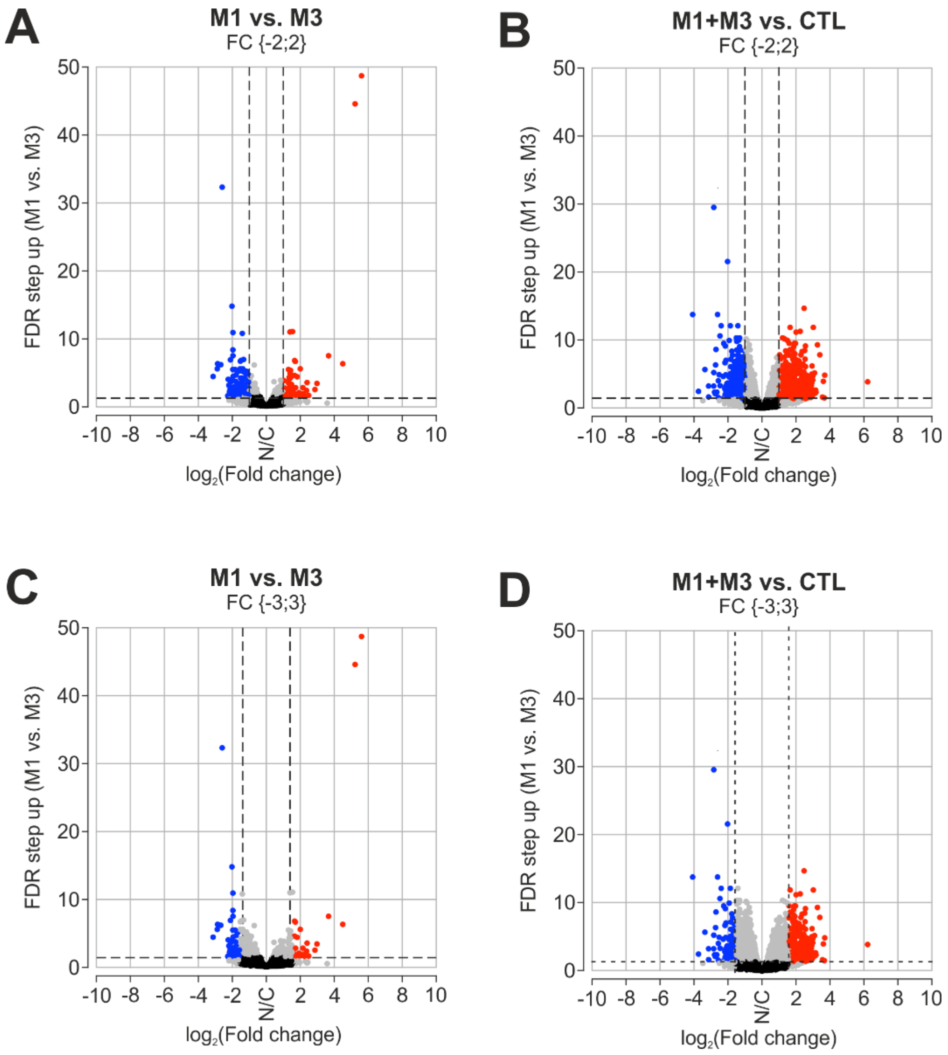

Fig. S3. RNAseq reveals few differences between NF1-mutant and CTL hiMGL cells

A Volcano plot demonstrating genes differentially expressed between M1 versus M3 [FDR < .05; fold change (-2, 2)]. Grey dots (no change), blue dots (decreased expression), red dots (increased expression

B Volcano plot demonstrating genes differentially expressed between M1 and M3 versus CTL [FDR < .05; fold change (-2, 2)]. Grey dots (no change), blue dots (decreased expression), red dots (increased expression).

C Volcano plot demonstrating genes differentially expressed between M1 versus M3 [FDR < .05; fold change (-3, 3)]. Grey dots (no change), blue dots (decreased expression), red dots (increased expression).

D Volcano plot demonstrating genes differentially expressed between M1 and M3 versus CTL [FDR < .05; fold change (-3, 3)]. Grey dots (no change), blue dots (decreased expression), red dots (increased expression).

Disease Models & Mechanisms • Supplementary information

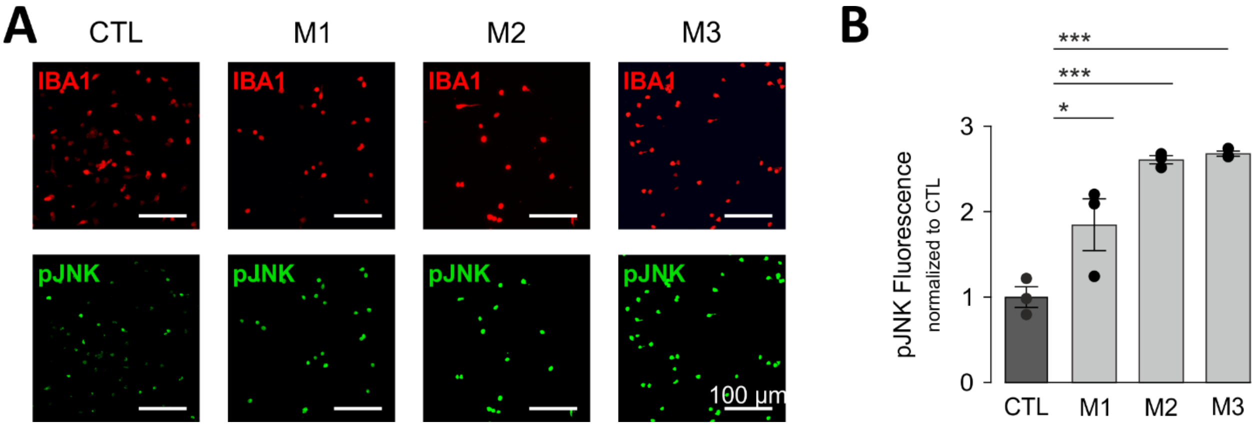

Fig. S4. *NF1*-mutant hiMGL cells show higher p-JNK expression than CTL hiMGL cells

A Immunohistochemical staining of CTL and *NF1*-mutant (M1-M3) hiMGL cells for IBA1 (red, *top*) and phosphorylated (activated) JNK (phospho-Thr^{183}/Tyr^{185} JNK, p-JNK; green, *bottom*). Scale bars, 100 µm.

B Quantification of phospho-JNK (p-JNK) fluorescence intensity in M1, M2 and M3 hiMGL cells, normalized to CTL hiMGL cells. Data indicated by asterisks were analyzed using a one-way ANOVA followed by Tukey's multiple comparisons test. *P<0.05; ***P<0.001. Results are represented as the mean +/- SEM. N = 3 independent differentiations for each mutant.

Disease Models & Mechanisms • Supplementary information

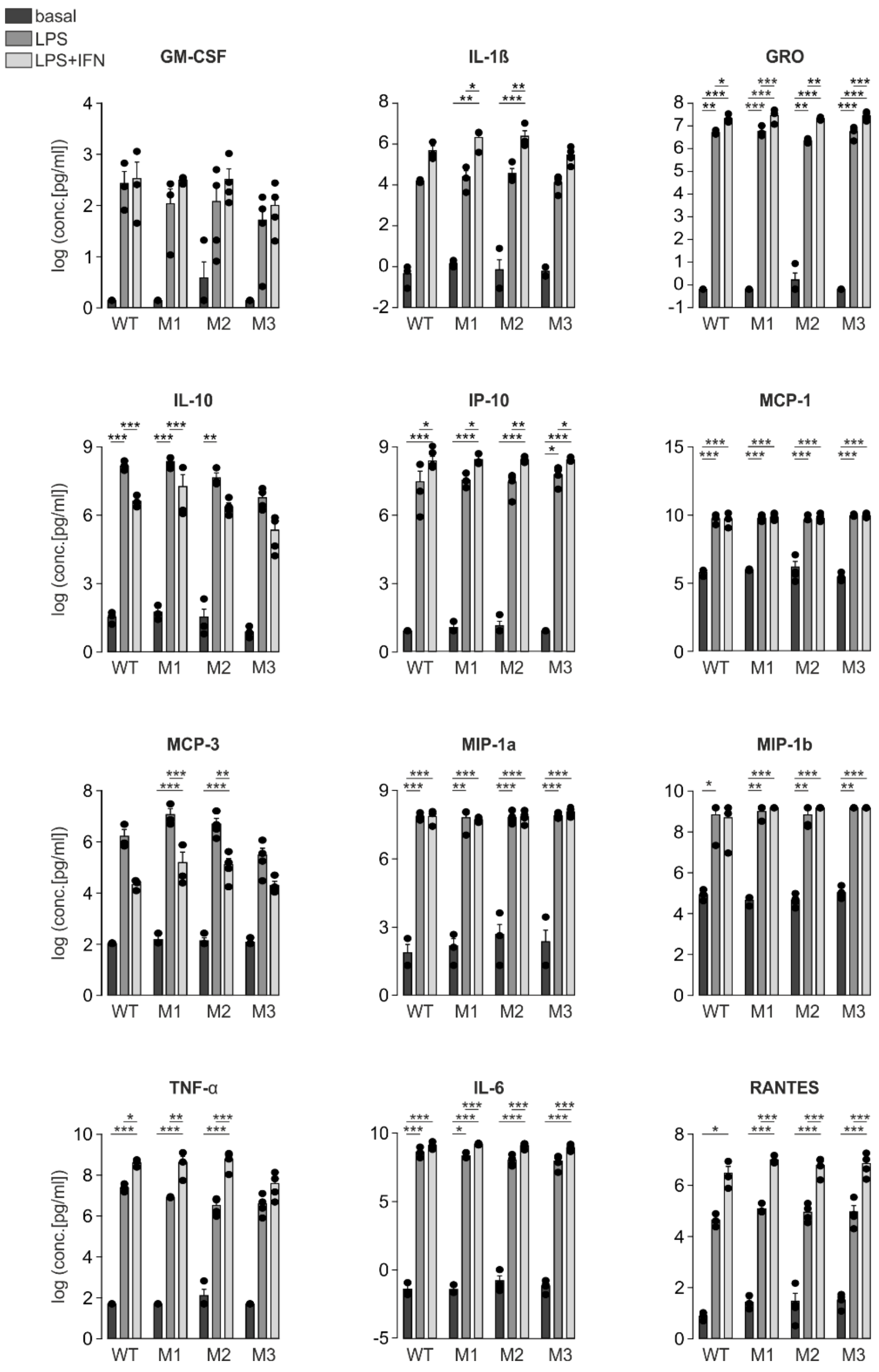

Fig. S5. Cytokine release from CTL and *NF1*-mutant hiMGL cells

A multiplex immunoassay was used to detect cytokines/chemokines secreted into the tissue culture medium by CTL and *NF1*-mutant hiMGL cells in response to stimulation with 1 µg/ml LPS or 1 µg/ml LPS + 100 ng/ml IFN-γ for 24h (n = 3-4). Data were analyzed by an ANOVA test. *P<0.05; **P< 0.01, ***P<0.001. Results are represented as the mean ± SEM.

Disease Models & Mechanisms • Supplementary information

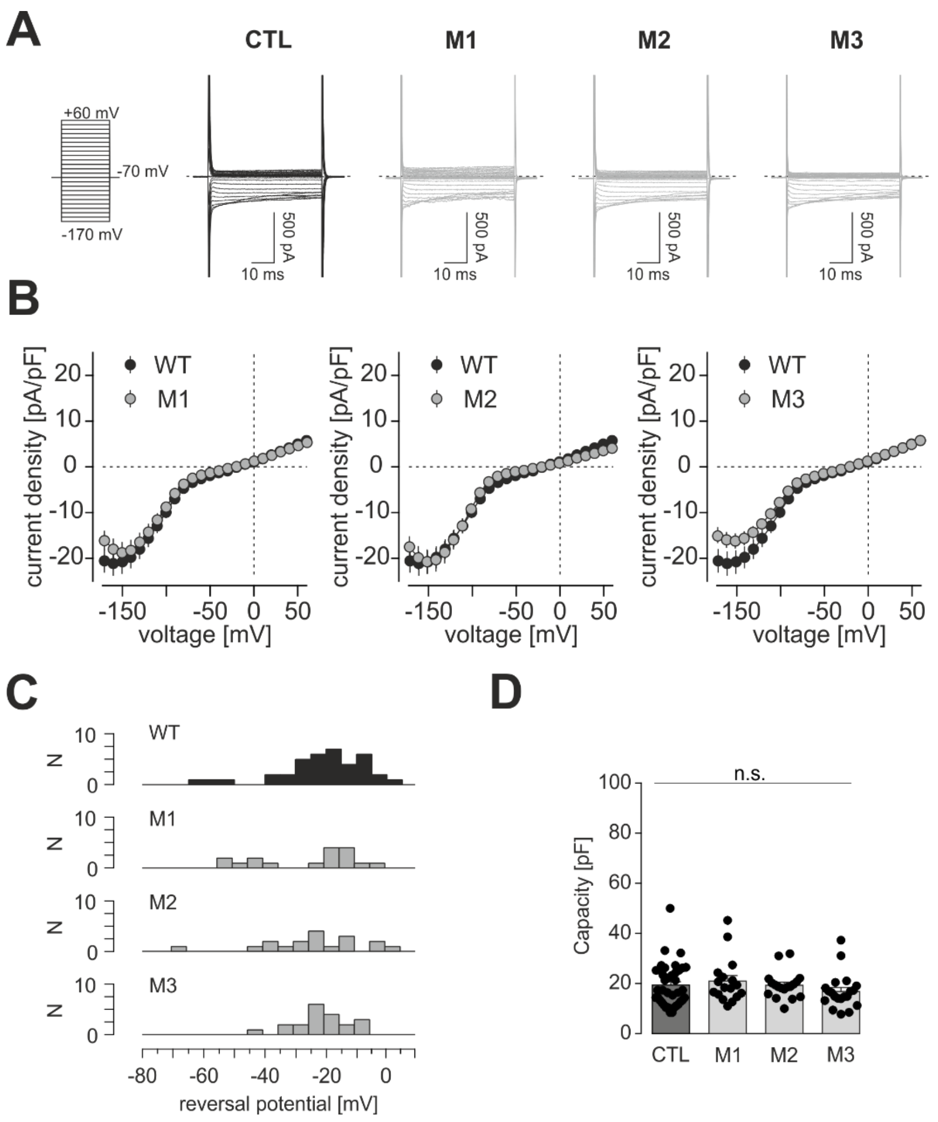

Fig. S6. Basal membrane properties of CTL and *NF1*-mutant hiMGL cells

A Sample patch clamp recordings of CTL and *NF1*-mutant hiMGL cells. Membrane currents were obtained during a series of voltage steps for 50 ms ranging from -170 mV to +60 mV from a holding potential of -70 mV.

B Average current density-voltage relationships of hiMGL cells obtained from the recordings shown in **A**.

C Distribution of the reversal potentials (indicative of the membrane potentials). N: number of patched cells.

D: Summary of the membrane capacities of CTL and *NF1*-mutant hiMGL cells.

Disease Models & Mechanisms • Supplementary information

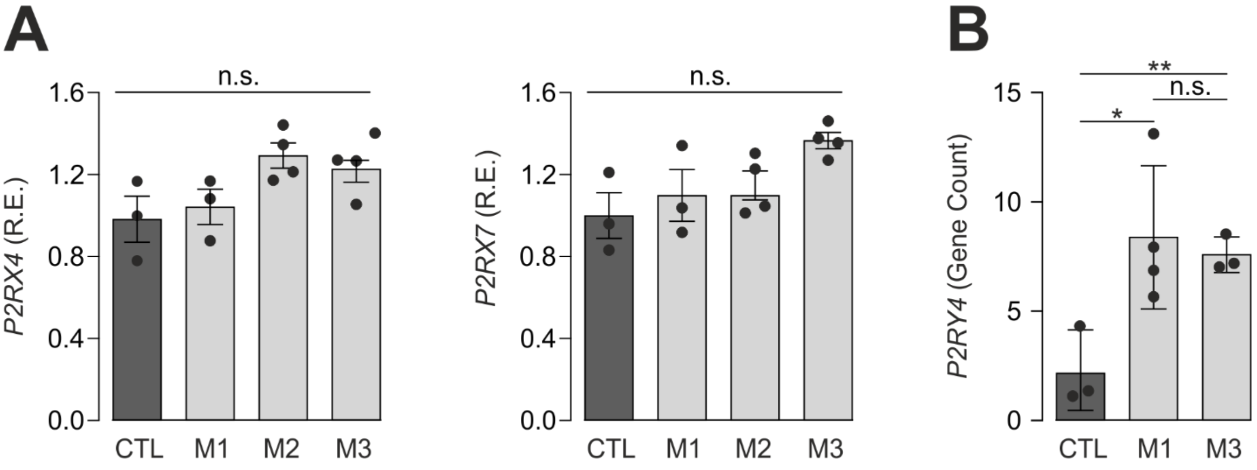

Fig. S7. Expression of P2RX4 and P2RX7 in CTL and *NF1*-mutant hiMGL cells

A: Relative *P2RX4* (left) and *P2RX7* mRNA expression (right) levels in CTL and *NF1*-mutant hiMGL cells by quantitative RT-PCR. TATA box binding protein (*TBP*) was used as a housekeeping gene for normalization (n = 3). Data were then normalized to mRNA expression levels in CTL hiMGL cells. Results are represented as the mean ± SD. Data were analyzed by one-way ANOVA.

B: P2 receptor expression from the RNA sequencing data revealed that only *P2RY4* expression was different in *NF1*-mutant hiMGL cells relative to CTL hiMGL cells. P values are included in the graph. All other genes were not statistically different between *NF1*-mutant and CTL hiMGL cell groups.

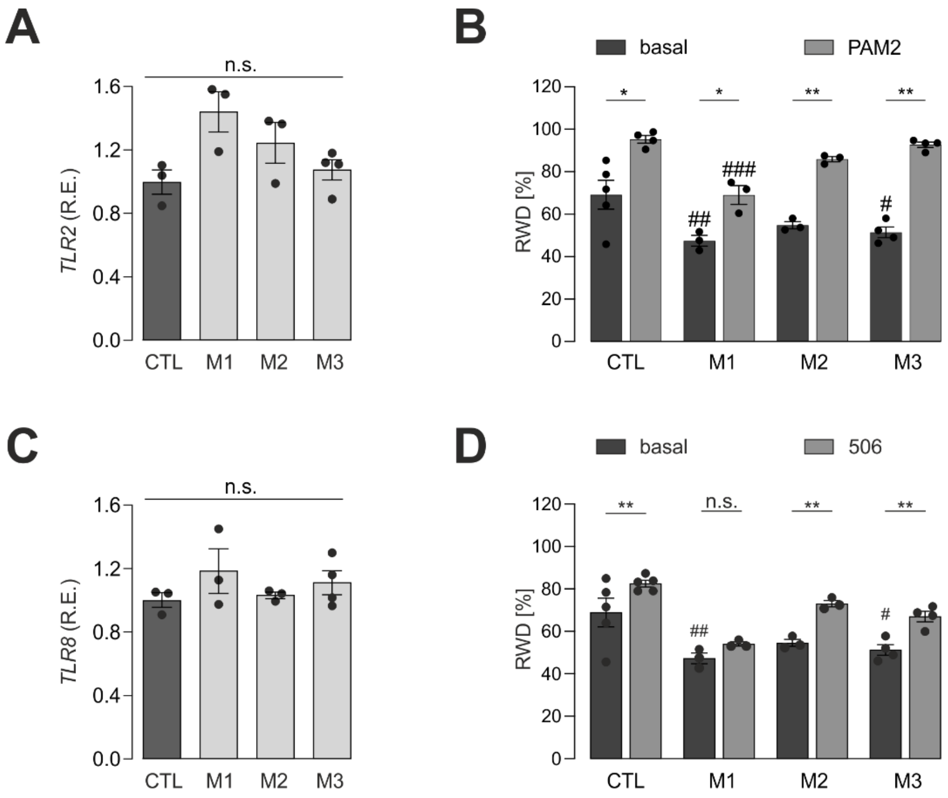

Fig. S8. Motility of CTL and *NF1*-mutant hiMGL cells

A Relative mRNA expression levels of Toll-like receptor 2 (*TLR2*) in CTL and *NF1*-mutant hiMGL cells by quantitative RT-PCR. Relative expression (R.E.) is shown relative to CTL hiMGL cells. TATA box binding protein (*TBP*) mRNA expression was used as a housekeeping gene for normalization (n = 3). Data were then normalized to *TLR2* expression of CTL hiMGL cells. Results are represented as the mean ± SEM. Data were analyzed by one-way ANOVA.

B Motility was assessed using a standardized wound scratch assay (RWD) using an Incucyte Zoom System. hiMGL cells were incubated with or without Pam2CSK4 (100 ng/ml). Relative Wound Density (RWD) was assessed over the course of two days comparing CTL and *NF1*-mutant hiMGL cells. Results are represented as the mean ± SEM. N = 5 for CTL and N = 3-4 for *NF1*-mutant hiMGL cells. Data indicated by the asterisks were analyzed by one-way ANOVA followed by Tukey's multiple comparisons test. *P<0.05; **P< 0.01, ***P<0.001. Comparisons between basal and Pam2CSK4 conditions were performed using a Student's t-test: #P<0.05, ###P<0.001.

Disease Models & Mechanisms • Supplementary information

C Relative mRNA expression levels of Toll-like receptor 8 (*TLR8*) was assessed in CTL and *NF1*-mutant hiMGL cells by quantitative RT-PCR. TATA box binding protein (*TBP*) was used as a housekeeping gene for normalization (n=3). Data were then normalized to *TLR8* expression of CTL hiMGL cells. Results are represented as the mean ± SD. Data were analyzed by one-way ANOVA.

D RWD after 36h was represented as the mean ± SEM. N = 5 for CTL and N = 3 for *NF1*-mutant hiMGL cells. hiMGL cells were incubated with or without 506 (100 ng/ml). Data indicated by the asterisks were analyzed by one-way ANOVA followed by Tukey's multiple comparisons test. *P<0.05; **P< 0.01, ***P<0.001. Comparisons between basal and 506 conditions were performed using a Student's t-test: ##P<0.01.

Disease Models & Mechanisms • Supplementary information

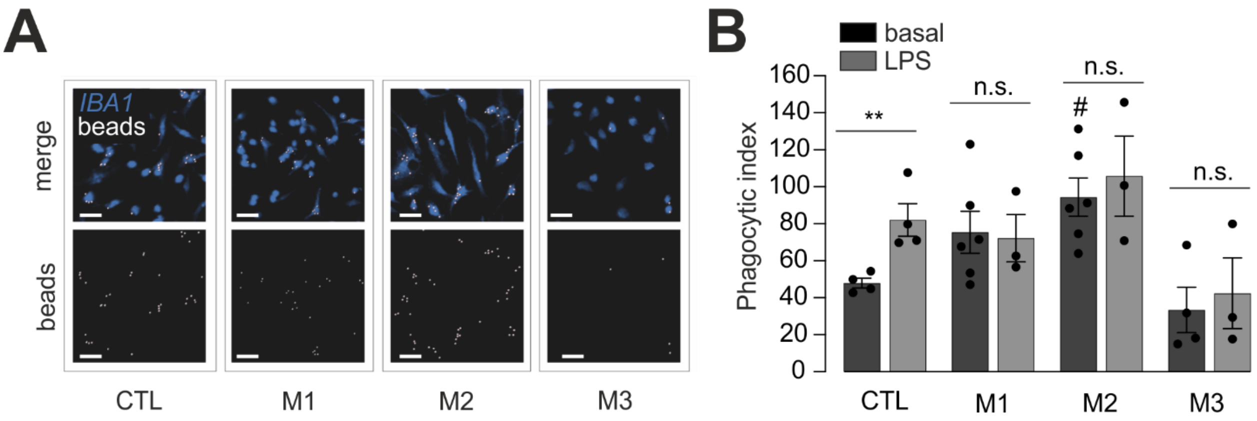

Fig. S9. Phagocytic activity of CTL and *NF1*-mutant hiMGL cells

Phagocytic activity was assessed by microscopy using fluorescent microbeads. CTL and *NF1*-mutant hiMGL cells were incubated for 1h with beads with or without addition of 1 µg/ml LPS.

A: Representative images of CTL and *NF1*-mutant hiMGL cells at the end of the assay period under LPS stimulation conditions. IBA1 staining is indicated in blue and beads in white. Scale bars, 20 µm.

B: Phagocytic activity presented as the phagocytic index, which is a measure of the percentage of cells harboring 0, 1, 2 or >3 engulfed beads. Data indicated by asterisks were analyzed by one-way ANOVA followed by Tukey's multiple comparisons test. *P<0.05; **P< 0.01, ***P<0.001. Comparisons between basal and UDP conditions were performed using a Student's t-test: ###P<0.001. Results are represented as the mean ± SEM. N = 5 for CTL and n = 3 for *NF1*-mutant hiMGL cells.

Disease Models & Mechanisms • Supplementary information

Table S1. Antibodies used

Antibody	Company	Host species	Dilution	Catalog number
Anti-IBA1	Abcam	Goat	1:250	ab5076
Anti-NANOG	Thermo Fisher Scientific	Rabbit	1:100	PA1-097
Anti Phospho-JNK (phospho-T183/Y185)	R&D Systems	Rabbit	1:100	MAB1205
Anti-OCT-3/4	Santa Cruz	Rabbit	1:100	sc-9081
Anti-P2RY12	Genetex	Rabbit	1:200	GTX54796
Anti-SSEA-4	Abcam	Mouse	1:100	ab16287
Anti-TMEM119	Abcam	Rabbit	1:200	ab185333
Anti-TRA-1-60	Abcam	Mouse	1:100	ab16288
Anti-Goat-AF488	Dianova	Donkey	1:200	705-545-147
Anti-mouse-AF647	Dianova	Donkey	1:125	715-605-151
Anti-rabbit-AF647	Dianova	Donkey	1:200	711-605-152
Anti-Goat-AF647	Dianova	Donkey	1:200	705-605-147

Disease Models & Mechanisms • Supplementary information

Table S2. Primers used

Primer		Sequence
AIF1	forward	5’- TTGGTGAGAAACGGGTGATTTG-3’
	reverse	5’- ATGGAGCATGTAGGAGAGACC-3’
P2RX4	forward	5’- GAGATTCCAGATGCGACCACT-3’
	reverse	5’- ACCCGTTGAAAGCTACGCAC-3’
P2RX7	forward	5’- TATGAGACGAACAAAGTCACTCG-3’
	reverse	5’- GCAAAGCAAACGTAGGAAAAGAT-3’
P2RY6	forward	5’- GTGTCTACCGCGAGAACTTCA-3’
	reverse	5’- CCAGAGCAAGGTTTAGGGTGTA-3’
P2RY12	forward	5’- CACTGCTCTACACTGTCCTGT-3’
	reverse	5’- AGTGGTCCTGTTCCCAGTTTG-3’
TBP1	forward	5’- AGCGCAAGGGTTTCTGGTTT-3’
	reverse	5’- CTGAATAGGCTGTGGGGTCA-3’
TLR2	forward	5’- TTATCCAGCACACGAATACACAG-3’
	reverse	5’- AGGCATCTGGTAGAGTCATCAA-3’
TLR4	forward	5’- TGGAAGTTGAACGAATGGAATGTG-3’
	reverse	5’- ACCAGAACTGCTACAACAGATACT-3’
TLR8	forward	5’- CCACCTTGAAGAGAGCCGAG-3’
	reverse	5’- TGCTCTGCATGAGGTTGTCG-3’
TMEM119	forward	5’- GAGGAGGGACGGGAGGAG-3’
	reverse	5’- CAGAAGGATGAGGAGGCTGG-3’

Disease Models & Mechanisms • Supplementary information

Table S3. Differentially expressed genes between M1 & M3 relative to CTL hiMGL cells

Gene name	P-value	FDR	Fold change (M1 & M3 vs. CTL)
DCANP1	6.18E-06	1.82E-04	71.76
GPR22	2.13E-05	4.53E-04	15.36
FOXN3-AS2	3.54E-10	1.07E-07	12.56
EHF	7.92E-05	1.18E-03	9.39
SAA1	7.42E-04	6.31E-03	9.31
YY1P1	5.45E-12	3.98E-09	9.00
RNF43	3.96E-03	2.18E-02	8.81
KCNAB1	2.76E-06	1.00E-04	7.57
ZNF22-AS1	2.83E-05	5.54E-04	7.44
CDH1	8.37E-04	6.93E-03	7.18
DSP	7.36E-05	1.12E-03	7.01
FAM83B	1.49E-03	1.06E-02	6.99
TAS2R30	5.23E-05	8.71E-04	6.65
OR7E38P	7.98E-06	2.17E-04	6.56
GAS8	6.93E-03	3.28E-02	6.54
KLB	2.36E-03	1.47E-02	6.36
OGN	1.29E-07	1.00E-05	6.32
EIF1P7	7.56E-03	3.51E-02	6.11
PLGLB2	6.39E-07	3.33E-05	6.02
LBH	1.08E-03	8.39E-03	5.95
PLN	2.00E-05	4.35E-04	5.94
LAMP3	9.26E-04	7.43E-03	5.90
PRMT5-AS1	7.23E-04	6.20E-03	5.76
SRGAP2-AS1	1.77E-05	3.99E-04	5.70
GNPDA2	3.21E-07	1.97E-05	5.45
PIGAP1	1.63E-06	6.66E-05	5.23
TAS2R13	4.79E-05	8.22E-04	5.22
DIAPH1-AS1	4.10E-09	7.42E-07	5.21
HSBP1L1	7.00E-03	3.30E-02	5.18
CCL22	7.69E-05	1.16E-03	5.18
CTXND1	8.63E-03	3.87E-02	5.16
CD1A	1.19E-08	1.68E-06	5.16
ATP5PDP4	2.24E-06	8.42E-05	5.13
TAS2R31	5.12E-04	4.81E-03	5.12
OR10A2	6.28E-04	5.59E-03	5.09
MANEA-DT	5.40E-03	2.72E-02	5.08
HSPD1P11	1.56E-03	1.09E-02	5.02
TMEM178A	1.61E-03	1.12E-02	-5.20
SAP25	2.52E-18	9.56E-15	-5.28
DNAH8	3.41E-04	3.57E-03	-5.39
PDCD6-AHRR	1.73E-14	4.11E-11	-5.80

Disease Models & Mechanisms • Supplementary information

TNFSF9	3.42E-06	1.18E-04	-5.94
SYNPO2L-AS1	6.61E-08	6.03E-06	-5.94
CAT	1.70E-20	1.08E-16	-6.39
DNAAF4-CCPG1	6.59E-06	1.90E-04	-6.39
GOPC	7.45E-27	1.21E-22	-6.90
MUSTN1	5.03E-04	4.77E-03	-7.80
CEMP1	7.07E-07	3.59E-05	-8.05
SDHAP2	6.63E-13	7.51E-10	-15.60

Table S4. Differentially expressed genes between M1 vs. M3 hiMGL cells

Gene name	P-value	FDR	Fold change (M1 vs. M3)
GOPC	7.08E-40	1.35E-35	46.33
ZNF252P	2.21E-33	2.10E-29	30.69
DNAAF4-CCPG1	6.18E-05	1.07E-02	10.40
MIR124-1HG	2.11E-04	2.39E-02	5.18
PCDHA10	3.09E-04	3.14E-02	5.04
BIRC3	2.29E-04	2.50E-02	-5.08
LTBP1	2.67E-08	2.21E-05	-5.96
PCDHGA10	1.66E-28	1.05E-24	-6.03
PRSS8	2.14E-09	2.55E-06	-6.69
TSSC2	4.48E-05	8.60E-03	-9.57
EHF	3.19E-11	5.05E-08	-11.10

Disease Models & Mechanisms • Supplementary information

Table S5. Materials for cell culture and *in vitro* experiments

Product name	Company	Catalog number
α-Thioglycerol	Merck	M1753-100ML
24-well plates	Sarstedt	83.3922
4′,6-diamidino-2-phenylindole (DAPI)	Merck	32670
Aqua-Poly/Mount	Polysciences Europe GmbH	18606
B-27 Supplement (50X), serum free	Life Technologies	17504-044
Bambanker	GC Lymphotec	302-14681
CD200, Human 50µg	Novoprotein	C311
CX3CL1, 5µg	Peprotech	300-31
Cyclic CAMP ELISA Kit	Cayman Chemical	581001
DMEM/F-12, HEPES, no phenol red	Life Technologies	11039-021
Donkey serum	Merck	S30
Falcon® 6-well Clear Flat Bottom Plates	Corning	353046
Fetal Calf Serum (FCS)	Life Technologies	10270-106
Fluoresbrite® YG Carboxylate Microspheres, 3.00µm	Polysciences Europe GmbH	17147-5
Geltrex™	Life Technologies	A14133-02
GlutaMAX Supplement-100 mL	Life Technologies	35050-038
HBSS	Life Technologies	14175-129
Human Insulin	PromoCell	C-52310
IL-34 2µg	Peprotech	200-34
Incucyte® Imagelock 96-well plates	Sartorius	BA-04856
Insulin-Transferrin-Selenite	Life Technologies	41400-045
Lipopolysaccharide from *E.coli*	Merck	L43191
M-CSF	Peprotech	300-25
MEM Non-Essential Amino Acids Solution (100X)-100 mL	Life Technologies	11140-035
TLR2 Agonist Pam2CSK4	R&D Systems	4637/1
P2RX4 Inhibitor 5-BDBD	R&D Systems	3579/10
P2RX7 Inhibitor A 740003	Tocris	3701
P2RY12 Inhibitor ARC-69931 Tetrasodium salt	Tocris, Bio-Techne GmbH	5720/1
N-2 Supplement (100X)	Life Technologies	17502-048
PBS	Life Technologies	14190-169
ReliaPrep™ RNA Tissue Kit	Promega	Z6112
STEMdiff™ Hematopoietic Kit	Stemcell Technologies	5310
StemMACS™ iPS-Brew XF	Miltenyi Biotec	130-107-086
StemPro® Accutase®	Life Technologies	A11105-01
SYBR Green Master Mix	Life Technologies	4472918
TGFβ1	Peprotech	100-21C
TL8-506	InvivoGen	Tlrl-Tl8506
Thiazovivin	StemCell Technologies™	72252
Triton® X-100	Roth	3051.3

Disease Models & Mechanisms • Supplementary information
